# UCP2 Identifies Immunosuppressive Tumor-Associated Macrophages and Is Associated with Predicted Immunotherapy Resistance in Glioma

**DOI:** 10.32604/or.2026.082613

**Published:** 2026-07-16

**Authors:** Hui Zhou, Jiarui Wang, Zhili Qiao, Xin Liao

**Affiliations:** 1 Department of Radiology, Affiliated Hospital of Guizhou Medical University, No. 28 Guiyi Street, Yunyan District, Guiyang, China; 2 Department of Radiology, The Affiliated Jinyang Hospital of Guizhou Medical University, No. 547 Jinyang South Road, Guanshanhu District, Guiyang, China; 3 Department of Pathology, Guiyang Maternal and Child Health Care Hospital, Guiyang, China; 4 Department of Neurosurgery, The Affiliated Hospital of Guizhou Medical University, No. 547 Jinyang South Road, Guanshanhu District, Guiyang, China

**Keywords:** UCP2, glioma, tumour-associated macrophages, tumour immune microenvironment, immune checkpoint, predicted immunotherapy resistance, single-cell RNA sequencing

## Abstract

Objectives: Uncoupling protein 2 (UCP2) has been extensively studied as a metabolic regulator in glioma; however, its relationship with the tumour immune microenvironment and the cellular source of its expression within the glioma tumour microenvironment (TME) remains poorly understood. This study aimed to characterise UCP2 expression at single-cell resolution and evaluate its immunological significance in glioma. Methods: This study employed an integrative multi-omics approach incorporating bulk transcriptomics, scRNA-seq (GSE70630, GSE84465, and GSE89567; n = 13,216 cells), immune deconvolution, immunohistochemistry (n = 96 glioma patients), and immunofluorescence co-staining (n = 6). Results: Pan-cancer analysis confirmed UCP2 overexpression in glioma. Single-cell analysis revealed UCP2 expression within the TME is predominantly enriched in macrophage/microglial populations rather than tumour cells, reframing UCP2 as a tumour-associated macrophage (TAM)-intrinsic regulator. In the TCGA cohort (n = 670), UCP2-high tumours exhibited an immunosuppressive landscape enriched with M2 macrophages and regulatory T cells, with elevated ESTIMATE scores. UCP2 correlated strongly with HAVCR2/TIM-3 (*ρ* = 0.847) and lactate metabolism genes, supporting a UCP2–lactate–TAM polarisation axis. TIDE analysis predicted that UCP2-high tumours may be associated with immunotherapy resistance. Multivariate Cox regression confirmed UCP2 as an independent prognostic factor (HR = 1.294, 95% CI: 1.116–1.502, *p* = 6.66 × 10^−4^). UCP2 protein expression negatively correlated with Ki67 (*ρ* = −0.446, *p* = 5.23 × 10^−6^), and immunofluorescence co-staining (n = 6) confirmed co-localisation with CD68-positive TAMs. Conclusion: UCP2 functions as a TAM-intrinsic immune regulator and candidate biomarker for immunosuppressive TME characterisation and immunotherapy response stratification in glioma.

## 1 Introduction

Glioma is the most common primary malignant tumor of the central nervous system in adults, accounting for the majority of intracranial malignancies [1]. Despite advances in multimodal treatment comprising surgical resection, radiotherapy, and temozolomide-based chemotherapy [2], patient prognosis remains dismal, with a five-year survival rate of less than 10% for high-grade glioma [2,3]. The immunosuppressive tumor microenvironment (TME) has emerged as a central determinant of treatment resistance and disease progression in glioma [4]. Unlike many other solid tumors that respond to immune checkpoint blockade, glioma is characterized by sparse cytotoxic T cell infiltration, abundant immunosuppressive tumor-associated macrophages (TAMs), and pervasive upregulation of inhibitory immune checkpoints, collectively rendering it refractory to current immunotherapeutic strategies [5]. Elucidating the molecular drivers of this immunosuppressive phenotype is therefore essential for identifying rational targets for immune sensitization.

Uncoupling protein 2 (UCP2), a member of the mitochondrial carrier superfamily, functions as a transporter of C4 metabolites—malate, oxaloacetate, and aspartate—across the inner mitochondrial membrane, thereby limiting glucose oxidation via the tricarboxylic acid (TCA) cycle and modulating intracellular reactive oxygen species (ROS) levels [6]. Tumor metabolic reprogramming, particularly aerobic glycolysis and lactate accumulation, is increasingly recognized as a key mechanism through which tumors remodel their immune landscape and evade immune surveillance [7,8], and UCP2-mediated suppression of mitochondrial glucose oxidation positions it as a potential upstream regulator of this immunosuppressive metabolic axis. Vallejo et al. [9,10]. demonstrated that UCP2 expression positively correlates with glioma grade and is associated with increased glycolytic dependence, while Wu et al. [11]. confirmed that UCP2 silencing suppresses glioma cell migration, invasion, and proliferation. More recently, Guo et al. [12]. conducted an integrative pan-cancer analysis of the UCP family and confirmed UCP2 as a prognostic indicator in glioblastoma, with experimental evidence linking UCP2 to epithelial-mesenchymal transition and radiation resistance. While these studies have established the metabolic and oncogenic dimensions of UCP2 in glioma, whether and how UCP2 shapes the immunosuppressive landscape of glioma—and critically, which cell populations drive its expression within the TME—remains unknown.

Using an integrative multi-omics approach combining bulk transcriptomics, single-cell RNA sequencing, immune deconvolution algorithms, functional enrichment analysis, and clinical validation, this study aimed to: (1) determine the predominant cellular source of UCP2 expression within the glioma TME; (2) investigate the biological functions and pathways associated with UCP2 expression through differential gene expression, functional enrichment, and gene set enrichment analyses; (3) systematically characterise the relationship between UCP2 expression and the immunosuppressive immune landscape, including immune cell composition, immune checkpoint expression, lactate metabolism, and TAM polarisation dynamics; (4) evaluate the independent prognostic value of UCP2 using survival analysis and multivariate Cox regression; and (5) validate UCP2 protein expression and its clinical correlates in an institutional glioma cohort comprising immunohistochemistry in 96 patients and immunofluorescence co-staining in a representative subset (n = 6), with the ultimate goal of establishing UCP2 as a candidate biomarker for immunosuppressive TME characterisation and immunotherapy response stratification in glioma.

## 2 Materials and Methods

### 2.1 Ethics Statement

This study was conducted in accordance with the Declaration of Helsinki. The use of clinical tissue samples from 96 glioma patients was approved by the Institutional Ethics Committee of The Affiliated Jinyang Hospital of Guizhou Medical University (approval number: JYYY-2025-XM-24). Written informed consent was waived by the Institutional Ethics Committee owing to the retrospective nature of the study and the use of anonymized archival tissue specimens.

### 2.2 Data Sources and Preprocessing

This study employed a retrospective cross-sectional design, integrating multi-source public transcriptomic data with institutional clinical validation. RNA-seq expression data for glioma patients were obtained from The Cancer Genome Atlas (TCGA) database (https://portal.gdc.cancer.gov/) [13]; all available low-grade glioma (LGG) and glioblastoma (GBM) samples were downloaded, and 702 samples were initially obtained; after quality control filtering retaining only primary tumour samples (sample code 01), 670 samples were retained for downstream analysis. Normal brain tissue expression profiles were downloaded from the Genotype-Tissue Expression (GTEx) database, encompassing all 13 available brain region subtypes (including cerebral cortex, cerebellum, hippocampus, and basal ganglia). A total of 1152 samples were retained after quality control filtering and used as a reference baseline for comparison with TCGA glioma tumour samples. No tissue-specific regional matching was performed (https://gtexportal.org/) [14], with 1152 brain tissue samples retained after quality filtering. For pan-cancer expression analysis, data from 31 cancer types were retrieved via the GEPIA platform (http://gepia.cancer-pku.cn/) [15], which integrates TCGA tumor and GTEx normal tissue data under a unified pipeline. All analyses were performed using R software (version 4.2.0, https://www.r-project.org/) [16]. TCGA RNA-seq data in Fragments Per Kilobase Million (FPKM) format were log_2_-transformed [log2(FPKM + 1)] to approximate normality. GTEx data, originally processed as log_2_(FPKM + 0.001), were back-transformed and re-normalized to the same log_2_(FPKM + 1) scale to ensure cross-dataset comparability prior to integrated analysis.

### 2.3 Single-Cell RNA Sequencing Analysis

To investigate the cell-type-specific expression of UCP2 in glioma, three publicly available scRNA-seq datasets were retrieved from the Gene Expression Omnibus (GEO) database (https://www.ncbi.nlm.nih.gov/geo/) [17]: GSE70630 (6 patients, 3304 cells, IDH-mutant oligodendroglioma), GSE84465 (4 patients, 3571 cells, GBM), and GSE89567 (10 patients, 6341 cells, IDH-mutant astrocytoma), encompassing a total of 13,216 cells across GBM and isocitrate dehydrogenase (IDH)-mutant glioma subtypes. Raw expression matrices were processed using the Seurat package (v 5.4.0) in R [18,19]. Quality control filtering retained cells expressing a minimum of 200 genes, and genes detected in fewer than 3 cells were excluded. Following log-normalization (Normalize Data; normalization.method = “LogNormalize”, scale.factor = 10,000), the top 2000 highly variable genes were identified (Find Variable Features; selection.method = “vst”, nfeatures = 2000) and used for downstream dimensionality reduction. Data were scaled (Scale Data), and principal component analysis (PCA) was performed with 30 components (npcs = 30), and the top 20 principal components were used for cell clustering and Uniform Manifold Approximation and Projection (UMAP) visualization [20]. Cell clustering was conducted using a shared nearest-neighbor graph (Find Neighbors, dims = 1:20) followed by the Louvain algorithm (Find Clusters, resolution = 0.5) [21], and UMAP was used for two-dimensional visualization. Cell type annotation was performed by examining the expression of established marker genes across clusters: CD14, AIF1, and CX3CR1 for macrophages/microglia; MBP, MOG, and PLP1 for oligodendrocytes; GFAP, VIM, and SOX2 for tumor cells; PECAM1 and VWF for endothelial cells; and TOP2A and MKI67 for proliferating cells. UCP2 expression across annotated cell populations was visualized using violin plots, and average expression values per cell type were calculated using the Average Expression function.

### 2.4 CCLE-Based Expression Analysis of UCP2 in Glioma Cell Lines

To examine baseline UCP2 expression in glioma tumour cells in the absence of microenvironmental influences, UCP2 expression data were retrieved from the Cancer Cell Line Encyclopedia (CCLE) via the DepMap portal (24Q4 release; https://depmap.org) [22]. RNA-seq libraries were sequenced on the Illumina NovaSeq platform. Preprocessed Log_2_(TPM + 1)-transformed expression values were directly retrieved from the DepMap portal (OmicsExpressionTPMLogp1HumanProteinCodingGenes) without further normalization. UCP2 expression values were extracted for all glioma cell lines, defined as those annotated with “Adult-Type Diffuse Glioma”, “Diffuse Glioma”, or related subtypes in the OncotreePrimaryDisease field of the DepMap Model metadata. A total of 74 unique glioma cell lines were identified after removing duplicates. Expression values were summarised descriptively and visualised using ggplot2.

### 2.5 Differential Expression and Functional Enrichment Analysis

Samples were stratified into UCP2-high and UCP2-low groups based on the median expression level. Differentially expressed genes (DEGs) between groups were identified using the limma package (v3.66.0) [23], with thresholds of |log_2_ fold change (FC)| > 1 and adjusted *p* value (*p*.adj) < 0.05. Multiple testing correction was performed using the Benjamini–Hochberg (BH) method to control the false discovery rate (FDR).

Gene Ontology (GO) biological process enrichment analysis and Kyoto Encyclopedia of Genes and Genomes (KEGG) pathway enrichment analysis of the DEGs were performed using the clusterProfiler package (v4.18.4) [24]. Statistical significance was defined as *p*.adj < 0.05. For pathways with fewer than 50 background genes, results were interpreted with caution owing to potential inflation of the Rich Factor metric.

GO biological process terms were classified as immune-related if the term itself was GO:0002376 (“immune system process”) or was annotated as a descendant of GO:0002376 based on “is_a” and “part_of” relationships in the GO directed acyclic graph (DAG). Ancestor–descendant relationships were retrieved using the GOBPANCESTOR mapping object from the GO.db Bioconductor package (v3.18) [25,26]. KEGG pathway classification was performed based on the official KEGG BRITE hierarchical system. The CLASS attribute for each enriched pathway was retrieved using the KEGGREST Bioconductor package (v1.50.0). Pathways with a CLASS field matching “Immune system” or “Immune disease” were defined as immune-related, and those under the “Metabolism” top-level category as metabolic [27].

Gene Set Enrichment Analysis (GSEA) was performed using the fgsea R package (v1.37.0), based on the GSEA analytical strategy [28]. All expressed genes were ranked by log_2_ FC derived from the limma differential expression analysis between UCP2-high and UCP2-low groups. The GO Biological Process gene set collection from the Molecular Signatures Database (MSigDB; c5.go.bp.v2025.1, Homo sapiens) was used as the reference gene set library [29]. Permutation testing was performed with 10,000 permutations (nperm = 10,000). Gene sets with a Benjamini–Hochberg-adjusted *p*-value (*p*.adj) < 0.05 were considered significantly enriched.

### 2.6 Protein–Protein Interaction Network Construction

A protein–protein interaction (PPI) network was constructed using the STRING database (version 11.5, https://string-db.org/) [30] with a minimum interaction confidence threshold of 0.4 (medium confidence). Network visualization was performed in Cytoscape (version 3.9.1, https://cytoscape.org) The STRING-derived network comprised 191 nodes and 979 edges. As only genes with at least one interaction meeting the minimum confidence threshold of 0.4 were retained by STRING, no isolated nodes were present and no additional topology filtering was applied in Cytoscape [31,32]. Hub genes within the network were identified using the Maximal Clique Centrality (MCC) algorithm implemented in the cytoHubba (v0.1) plugin.

### 2.7 Immune Microenvironment Analysis

#### 2.7.1 CIBERSORT Immune Cell Deconvolution

The relative proportions of 22 immune cell subtypes were estimated using the CIBERSORT algorithm (https://cibersortx.stanford.edu/) [33]. The normalized gene expression matrix was submitted to the CIBERSORT platform using the LM22 immune cell signature matrix (a 547-gene reference matrix of 22 human haematopoietic cell phenotypes), with 1000 permutations. Only samples with a CIBERSORT output *p* value < 0.05 were retained for downstream analysis to ensure reliability of deconvolution results. Differences in immune cell proportions between UCP2-high and UCP2-low groups were assessed using the Wilcoxon rank-sum test across all 22 immune cell subsets. Multiple testing correction was applied using the Benjamini–Hochberg (BH) method. Given the exploratory nature of this analysis, cell types with raw *p* < 0.05 were considered as nominally significant.

#### 2.7.2 ESTIMATE Algorithm

TME composition was further characterized using the ESTIMATE algorithm (estimate R package, v1.0.13). Log2(FPKM + 1)-transformed gene expression profiles from the TCGA glioma cohort were used as input. This method infers infiltration levels of immune and stromal cells from gene expression profiles, yielding three scores: the Immune Score, the Stromal Score, and the ESTIMATE Score [34]. This method infers infiltration levels of immune and stromal cells from gene expression profiles, yielding three scores: the Immune Score (reflecting immune cell infiltration), the Stromal Score (reflecting stromal cell content), and the ESTIMATE Score (the sum of the two, inversely related to tumor purity). Spearman correlation analysis was used to assess associations between UCP2 expression and each score.

#### 2.7.3 Immune Checkpoint Correlation Analysis

The expression of nine key immune checkpoint genes was extracted from the TCGA glioma cohort: PDCD1 (PD-1), CD274 (PD-L1), CTLA4, LAG3, HAVCR2 (TIM-3), TIGIT, IDO1, CD47, and CD276 (B7-H3). Spearman correlation analysis was performed to evaluate associations between UCP2 expression and each checkpoint gene. Multiple testing correction was applied using the Benjamini–Hochberg (BH) method, and adjusted *p* values (*p*.adj) were used to determine statistical significance. Correlation strength was classified according to Cohen’s criteria: |*r*| < 0.3 (weak), 0.3 ≤ |*r*| < 0.7 (moderate), and |*r*| ≥ 0.7 (strong).

#### 2.7.4 TIDE Analysis

Immunotherapy response prediction was performed using the Tumor Immune Dysfunction and Exclusion (TIDE) algorithm (http://tide.dfci.harvard.edu/) [35]. TIDE evaluates two dimensions: Dysfunction (reflecting T cell exhaustion within the tumor) and Exclusion (reflecting impediment of T cell infiltration). A higher TIDE score indicates greater likelihood of immune evasion and lower probability of immunotherapy benefit. Spearman correlation between UCP2 expression and TIDE scores was calculated separately for the overall TIDE score, the Dysfunction score, and the Exclusion score. Given the limited number of comparisons (n = 3), no multiple testing correction was applied.

### 2.8 Correlation Analysis between UCP2, Lactate Metabolism-Related Genes, Macrophage Infiltration Markers, and Assessment of Tumour Purity Confounding

To investigate the relationship between UCP2 expression and tumour glycolytic reprogramming, Spearman correlation analysis was performed between UCP2 and seven key lactate metabolism-related genes—SLC16A1 (MCT1), SLC16A3 (MCT4), LDHA, LDHB, HK2, PKM2, and SLC2A1 (GLUT1)—across TCGA glioma samples (n = 670). These genes were selected based on their established roles in key steps of tumour glycolytic reprogramming and lactate metabolism, encompassing glucose uptake (SLC2A1/GLUT1), rate-limiting glycolytic enzymes (HK2, PKM2), lactate production and interconversion (LDHA, LDHB), and lactate transport across the plasma membrane (SLC16A1/MCT1, SLC16A3/MCT4). Spearman’s rank correlation coefficients and corresponding *p*-values were calculated using the cor.test function in R (v4.2.0) (method = “spearman”). Multiple testing correction was applied using the Benjamini–Hochberg FDR method via *p*.adj. Results were visualised as scatter plots with linear regression fits using ggplot2 and assembled into a composite figure using ggpubr. Linear regression fits were included for visualization purposes to illustrate the overall trend direction, which is consistent with the monotonic relationships indicated by the Spearman correlation analysis. Statistical inference was based solely on Spearman’s rank correlation coefficients and their associated *p* values. Statistical significance was defined as *p*.adj < 0.05.

To assess the relationship between UCP2 expression and macrophage infiltration at the transcriptomic level, Spearman correlation analysis was additionally performed between UCP2 and CD68 mRNA expression across the same TCGA glioma cohort. Expression data were extracted from the normalised expression matrix, and Spearman’s rank correlation coefficient and corresponding *p*-value were calculated using the cor.test function in R (v4.2.0) (method = “spearman”). Results were visualised as a scatter plot with a linear regression fit and 95% confidence interval using ggplot2 [36].

To evaluate whether the UCP2–CD68 association could be attributable to differences in tumour cellularity rather than genuine macrophage infiltration, partial correlation analysis was performed using the pcor.test function from the ppcor R package (v1.1) (method = “spearman”), with ESTIMATEScore used as a proxy for tumour purity. Results were visualised as scatter plots using ggplot2 and assembled using ggpubr.

### 2.9 Co-Expression Analysis of UCP2 and HAVCR2 within Macrophage/Microglial Clusters in scRNA-seq Data

To determine whether UCP2 and HAVCR2 are co-expressed within the same macrophage/microglial populations, single-cell co-expression analysis was performed across the three independent scRNA-seq datasets (GSE70630, GSE84465, and GSE89567). To avoid potential batch effects, no dataset integration or batch effect correction was applied; instead, analyses were performed separately for each dataset within the isolated macrophage/microglial populations. For each dataset, processed Seurat objects were loaded and macrophage/microglial cells were isolated based on previously established cell-type annotations. Log-normalised expression values of UCP2 and HAVCR2 were extracted from the data slot of each Seurat object using GetAssayData (Seurat, v5.4.0). To avoid potential batch effects, no dataset integration or batch effect correction was applied; instead, Spearman correlation analysis was performed separately for each dataset and for the combined macrophage/microglial population across all three datasets using cor.test in R (v4.2.0) (method = “spearman”). As the correlation analysis addressed a single biological question (co-expression of UCP2 and HAVCR2) validated across independent datasets rather than testing multiple hypotheses simultaneously, no multiple testing correction was applied. Results were visualised as faceted scatter plots with per-dataset correlation annotations, and as a two-dimensional density plot for the combined cohort, using ggplot2.

### 2.10 Pseudotime Trajectory Analysis of Tumor-Associated Macrophages

To reconstruct the differentiation trajectory of tumor-associated macrophages (TAMs) and assess the relationship between UCP2 expression and TAM polarization state, pseudotime analysis was performed using the Slingshot algorithm (v2.18.0) [37]. TAMs (annotated as “Macrophage/Microglia”) were extracted from three independent glioma scRNA-seq datasets (GSE70630, GSE84465, and GSE89567) and merged into a unified object comprising 3074 cells. Data integration and standard preprocessing were performed using Seurat (v5.4.0) without formal batch effect correction. Standard preprocessing was subsequently applied to the merged object, including library-size normalization (NormalizeData), identification of the top 2000 highly variable features using the variance-stabilizing transformation method (FindVariableFeatures; selection.method = “vst”, nfeatures = 2000), scaling (ScaleData), and dimensionality reduction via PCA (30 components). The number of principal components for downstream analyses (dims = 1:20) was determined based on ElbowPlot inspection, which indicated that the majority of variance was captured within the first 20 principal components. UMAP visualization and unsupervised clustering were subsequently performed using a shared nearest-neighbor graph (FindNeighbors, dims 1–20) and the Louvain algorithm (FindClusters, resolution = 0.5).

The merged Seurat object was converted to a SingleCellExperiment object [38] and passed to slingshot (v2.18.0), with UMAP coordinates as the reduced dimension space and Seurat cluster labels as input. Where multiple lineages were inferred, the lineage exhibiting the strongest Spearman correlation with UCP2 expression was selected as the principal trajectory, Cells assigned to other lineages or receiving non-finite pseudotime values (NA or Inf), reflecting cells not assigned to the selected principal trajectory by the Slingshot algorithm, were excluded from downstream analysis. This resulted in 1575 TAMs with finite pseudotime assignments out of 3074 total merged TAMs (51.2% retained). M1 and M2 polarization module scores were computed using AddModuleScore with canonical marker gene sets: M1 markers (TNF, IL1B, IL6, NOS2, CD86, CXCL9, CXCL10, IRF1, STAT1) and M2 markers (CD163, MRC1, TGFB1, IL10, ARG1, CCL18, MSR1, FOLR2, MAF). Spearman rank correlation between pseudotime values and log-normalized gene expression (or module scores) was computed using cor.test in R (v4.2.0). As the pseudotime correlation analyses were exploratory in nature and involved a limited number of pre-specified genes and module scores, no multiple testing correction was applied. Locally estimated scatterplot smoothing (LOESS) smoothing curves were fitted to visualize expression dynamics along the inferred trajectory. All analyses were conducted in R (version 4.2.0) with visualization performed using ggplot2 (v4.0.2) and patchwork (v1.3.2, https://CRAN.R-project.org/package=patchwork).

### 2.11 Survival Analysis and Cox Proportional Hazards Regression

Overall survival (OS) data were obtained from the TCGA glioma clinical dataset via the GDC data portal (https://portal.gdc.cancer.gov/), accessed in October 2025. Patients were included if complete data were available for OS time, OS status, UCP2 expression, age, WHO grade, and IDH mutation status. Patients with missing values in any of these variables were excluded using complete case analysis. Patients were stratified into UCP2-high and UCP2-low groups based on the median UCP2 expression value. Survival analysis was performed using the survival R package (v3.8.6), and Kaplan–Meier (KM) survival curves were generated and visualised using the survminer package (0.5.2). Between-group differences in survival were assessed using the log-rank test. Univariate Cox proportional hazards regression was performed to evaluate the prognostic effect of UCP2 expression on OS. Multivariate Cox regression was subsequently conducted incorporating UCP2 expression, age, WHO grade, and IDH mutation status as covariates, to determine whether UCP2 is an independent prognostic factor. Hazard ratios (HR) with 95% confidence intervals (CI) and corresponding *p*-values were reported, and results were visualised as a forest plot using ggforest from the survminer package (v0.5.2). As the survival analyses comprised a single Kaplan–Meier analysis and a prespecified multivariate Cox regression model incorporating clinically established covariates, no additional correction for multiple comparisons was applied.

### 2.12 Immunohistochemistry

Formalin-fixed, paraffin-embedded (FFPE) tissue sections were retrospectively collected from glioma patients at The Affiliated Jinyang Hospital of Guizhou Medical University between January 2021 and July 2025. Patients were eligible if they had: (1) a histopathologically confirmed diagnosis of glioma according to the 2021 World Health Organization (WHO) Classification of Tumours of the Central Nervous System; (2) available archival FFPE tissue with sufficient quality for IHC staining; and (3) complete clinicopathological records including WHO grade, IDH status, p53 status, and Ki67 index. Patients were excluded if they had: (1) received prior radiotherapy or chemotherapy before surgical resection; or (2) insufficient tissue material for reliable IHC scoring. A total of 212 glioma patients were initially screened. After excluding 48 cases with insufficient FFPE tissue quality, 41 cases with incomplete clinicopathological data, and 27 cases who had received prior radiotherapy or chemotherapy before surgical resection, a final cohort of 96 patients was included in the analysis. No formal sample size calculation was performed; all consecutive eligible archival cases identified within the study period were included. Sections were deparaffinized, rehydrated, and subjected to antigen retrieval using sodium citrate buffer (pH 6.0) at 95°C for 20 min. Endogenous peroxidase activity was blocked with 3% hydrogen peroxide for 10 min at room temperature. Non-specific binding was blocked with 5% bovine serum albumin (BSA; Solarbio, cat. A8010, Beijing, China) for 30 min at room temperature. Sections were incubated overnight at 4°C with a rabbit polyclonal anti-UCP2 primary antibody (Proteintech, cat. 11081-1-AP, Wuhan, China; 1:150 dilution), followed by incubation with a horseradish peroxidase (HRP)-conjugated goat anti-rabbit secondary antibody (Beyotime, cat. A0208, Shanghai, China; 1:1000 dilution) for 1 h at room temperature. Visualization was performed using a 3,3′-diaminobenzidine (DAB) chromogen kit (Solarbio, cat. DA1010, Beijing, China), and sections were counterstained with Mayer’s hematoxylin (Solarbio, cat. G1080, Beijing, China). UCP2 protein expression was evaluated using the immunoreactive score (IRS), calculated as the mean product of staining intensity (0 = negative, 1 = weak, 2 = moderate, 3 = strong) and the proportion of UCP2-positive cells across five randomly selected minhigh-power fields, captured using a light microscope (Shanghai Cewi Optical Technology Co., Ltd., model LW370LT, Shanghai, China) at ×400 magnification. IRS values ranged from 0 to 3. Scoring was performed independently by two pathologists blinded to clinical information. Patients were stratified into UCP2-high and UCP2-low groups based on the median IRS value.

### 2.13 Immunofluorescence Co-Staining

To spatially confirm the co-localisation of UCP2 protein with tumour-associated macrophages at the tissue level, immunofluorescence (IF) dual-labelling was performed on FFPE tissue sections from a subset of six glioma specimens drawn from the clinical IHC cohort. Sections (2–3 μm) were baked at 65°C for 45 min to remove excess paraffin, then deparaffinised in xylene twice (30 min each), and rehydrated through a graded ethanol series: 100% ethanol twice (15 min each), 95% ethanol twice (15 min each), 85% ethanol (15 min), 75% ethanol twice (15 min each), and 50% ethanol twice (15 min each), followed by immersion in distilled water for 30 min. Antigen retrieval was performed using sodium citrate buffer (pH 6.0) in an electric pressure cooker (Midea Group Co., Ltd., model MY-YL50X3-102R, Foshan, China) at 121°C and 103 kPa for 2 min, then removed from heat and allowed to cool naturally. Non-specific binding was blocked with 5% bovine serum albumin (BSA; Solarbio, cat. A8010, Beijing, China) for 30 min at room temperature. Sections were incubated overnight at 4°C with a cocktail of two primary antibodies diluted in 1% BSA: a rabbit anti-UCP2 antibody (Affinity Biosciences, Jiangsu, China, cat. DF8626; 1:150 dilution) and a mouse anti-CD68 antibody (MedChemExpress, cat. HY-P80605, Shanghai, China; 1:150 dilution). After three washes with PBS (pH 7.4, 10 min each), sections were incubated for 2 h at room temperature in the dark with a mixture of fluorescence-conjugated secondary antibodies: CoraLite Plus 488-conjugated goat anti-rabbit secondary antibody (green; Proteintech, cat. RGAR002) for UCP2 detection, and CoraLite Plus 555-conjugated goat anti-mouse secondary antibody (yellow; Proteintech, cat. RGAM003) for CD68 detection. After three further phosphate-buffered saline (PBS) washes, cell nuclei were counterstained with 4′,6-diamidino-2-phenylindole (DAPI) (Solarbio, cat. C0065) for 10 min in the dark. Sections were mounted using anti-fade mounting medium (Solarbio, cat. S2110) and imaged using a fluorescence microscope (Cewi Optical Technology Co., Ltd., Shanghai, China, model LWD300-66LFT) at ×200 magnification. A minimum of three non-overlapping fields were captured per section.

### 2.14 Statistical Analysis

Continuous variables are expressed as median (interquartile range, IQR); categorical variables are expressed as frequency (percentage). Comparisons between two groups were performed using the Wilcoxon rank-sum test; comparisons among multiple groups used the Kruskal-Wallis test. Correlations between continuous variables were assessed by Spearman’s rank correlation coefficient. All statistical tests were two-tailed, and *p* < 0.05 was considered statistically significant. All statistical analyses were conducted in R (version 4.2.0). No missing data were identified for any of the clinicopathological variables included in the analysis; therefore, complete case analysis was performed.

## 3 Results

### 3.1 UCP2 Is Significantly Overexpressed in Pan-Cancer and Gliomas

UCP2 was significantly upregulated in 19 of 31 cancer types examined (*p* < 0.01), including both glioma subtypes—low-grade glioma (LGG) and glioblastoma (GBM)—which showed significant UCP2 overexpression relative to corresponding normal brain tissue (Supplementary Fig. S1). Further comparison between TCGA glioma samples (n = 670) and GTEx normal brain tissue (n = 1152) confirmed significant UCP2 overexpression in tumor tissue (Wilcoxon rank-sum test, *p* < 2 × 10^−^^16^; Supplementary Fig. S2).

### 3.2 UCP2 Is Predominantly Expressed in Macrophages and Microglia within the Glioma Microenvironment

**Figure 1:**
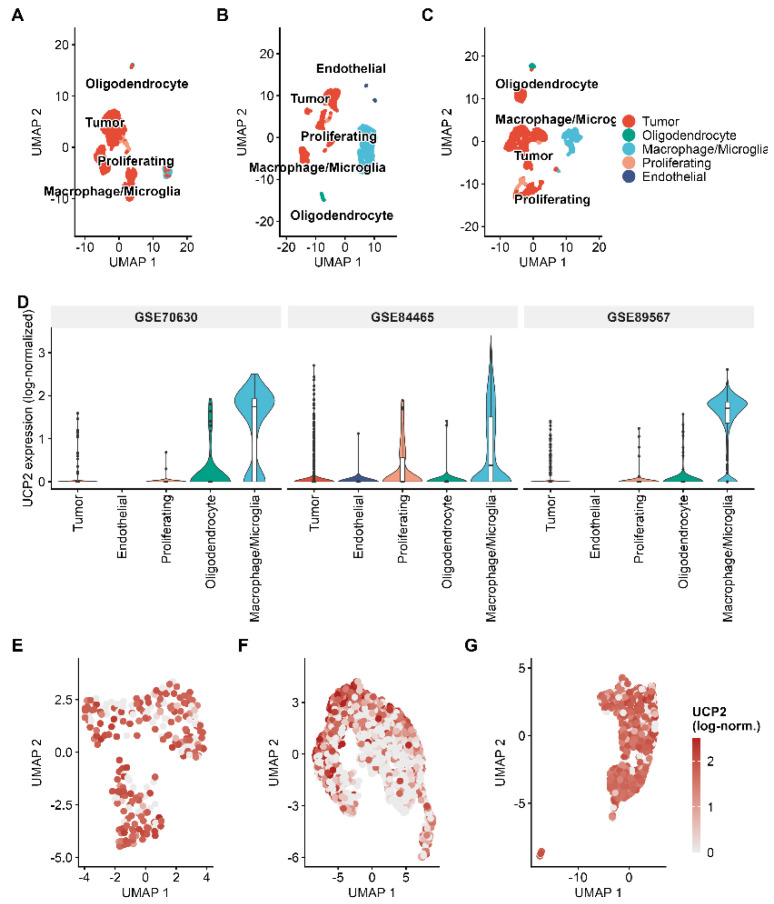
UCP2 expression is predominantly enriched in macrophage/microglial populations across independent glioma scRNA-seq datasets. (**A**–**C**) UMAP plots of major cell populations in GSE70630 (**A**), GSE84465 (**B**), and GSE89567 (**C**), coloured by annotated cell type. (**D**) Violin plots showing the distribution of UCP2 expression (log-normalised counts) across cell types in each dataset. (**E**–**G**) UMAP feature plots of UCP2 expression within the macrophage/microglial subpopulation in GSE70630 (**E**), GSE84465 (**F**), and GSE89567 (**G**). Colour intensity represents UCP2 expression level (log-normalised) from low (light grey) to high (dark red).

To determine the cellular source of UCP2 expression within the glioma tumour microenvironment, three independent scRNA-seq datasets were analysed: GSE70630 (n = 3304 cells), GSE84465 (n = 3571 cells), and GSE89567 (n = 6341 cells), comprising a total of 13,216 single cells derived from glioma patients. Raw count matrices were processed using the Seurat pipeline, incorporating normalisation, identification of highly variable features, principal component analysis (PCA), UMAP-based dimensionality reduction, and graph-based unsupervised clustering. Cell populations were manually annotated based on the expression of established lineage marker genes: *CD14*, *AIF1*, and *CX3CR1* for macrophages/microglia; *MBP*, *MOG*, and *PLP1* for oligodendrocytes; *GFAP*, *VIM*, and *SOX2* for tumour cells; *PECAM1* and *VWF* for endothelial cells; and *TOP2A* and *MKI67* for proliferating cells. Major cell populations identified across the three datasets included tumour cells, macrophages/microglia, oligodendrocytes, and proliferating cells, with endothelial cells additionally identified in GSE84465. Across all three independent datasets, UCP2 expression was consistently and predominantly enriched in macrophage/microglial populations relative to all other major cell types examined (Kruskal-Wallis: GSE70630, χ^2^ = 2038.3, df = 3; GSE84465, χ^2^ = 1097.8, df = 4; GSE89567, χ^2^ = 4843.5, df = 3; all *p* < 0.001; Fig. 1A–C). Post-hoc Dunn’s pairwise comparisons with Benjamini–Hochberg correction confirmed that macrophage/microglial cells exhibited significantly higher UCP2 expression than all other annotated cell populations across the combined cohort (χ^2^ = 7455.4, df = 4, *p* < 0.001; all pairwise adjusted *p* < 0.001; Fig. 1D). To further characterise the intra-population distribution of UCP2 within macrophage/microglial cells, UCP2 expression was visualised in macrophage/microglial subsets across all three datasets, revealing widespread and heterogeneous UCP2 expression throughout these populations (Fig. 1E–G).

### 3.3 UCP2 Is Detectably Expressed in Glioma Cell Lines in the Absence of Tumour Microenvironmental Influences

UCP2 expression was examined across 74 unique glioma cell lines in the CCLE dataset. UCP2 was detectably expressed in 73 of 74 cell lines (98.6%), with expression levels ranging from 0 to 8.38 log_2_(TPM + 1) and a median expression of 4.09 log_2_(TPM + 1). Detectable expression was observed across all major glioma subtypes represented in the dataset, including Glioblastoma IDH-Wildtype, Astrocytoma IDH-Mutant, and Gliosarcoma (Supplementary Fig. S3).

### 3.4 UCP2 High Expression Is Associated with Immune-Related Gene Programs

**Figure 2:**
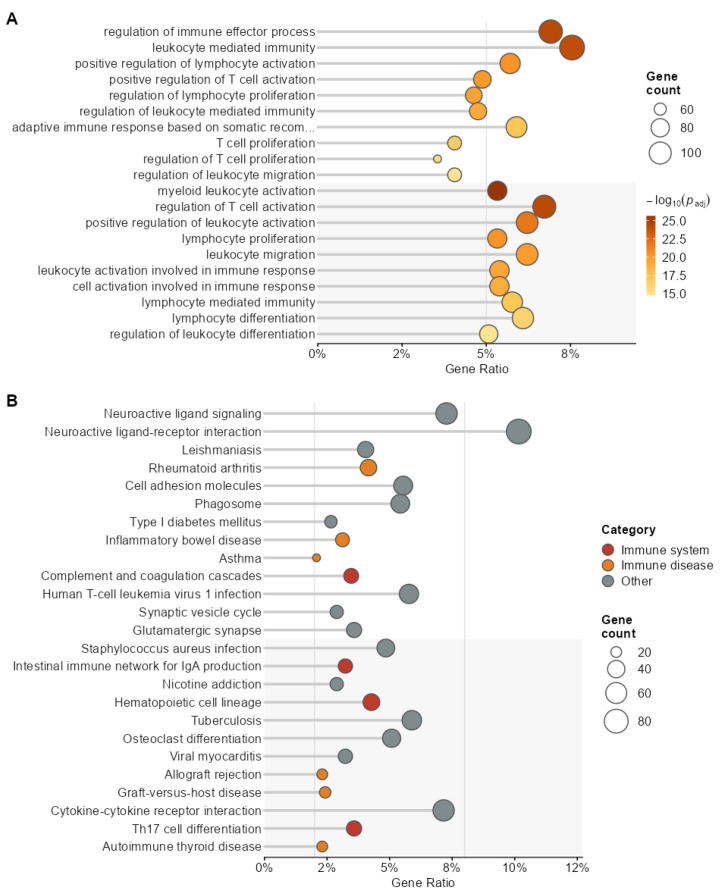
UCP2 high expression is associated with immune-related gene programs in glioma. (**A**) Bubble plot showing the top 20 immune-related Gene Ontology Biological Process (GO BP) terms enriched among differentially expressed genes (DEGs) between UCP2-high and UCP2-low groups (*p*.adj < 0.05). GO terms were classified as immune-related based on annotation as descendants of GO:0002376 (“immune system process”) in the GO directed acyclic graph. Bubble size represents the number of DEGs annotated to each term (gene count); bubble colour indicates statistical significance (−log_10_(*p*.adj), yellow to dark orange). Terms are ranked by gene ratio in descending order. (**B**) Bubble plot of the top 25 significantly enriched KEGG pathways (*p*.adj < 0.05), coloured by KEGG BRITE functional category: red, immune system pathways; orange, immune disease pathways; grey, other pathways. Bubble size represents gene count. Two amino acid metabolism pathways (tryptophan metabolism and phenylalanine metabolism) were significantly enriched but are not displayed as they ranked outside the top 25 pathways (see Supplementary Table S3).

Differential expression analysis was performed using the limma R package with thresholds of |log_2_FC| > 1 and FDR < 0.05, identifying 1926 differentially expressed genes (DEGs) between UCP2-high and UCP2-low groups, comprising 1140 upregulated and 786 downregulated genes (Supplementary Fig. S4A; Supplementary Table S1). Gene Ontology (GO) biological process enrichment analysis of the DEGs identified 1445 significantly enriched terms (*p*.adj < 0.05) involving 1299 unique genes. Using GO ontology-based classification (terms annotated as descendants of GO:0002376 “immune system process” in the GO directed acyclic graph), 378 terms (26.2%), involving 399 unique genes, were categorised as immune-related biological processes, including leukocyte activation, myeloid leukocyte function, T cell and lymphocyte regulation, leukocyte migration, and lymphocyte proliferation (Fig. 2A; Supplementary Table S2). KEGG pathway analysis identified 96 significantly enriched pathways (Supplementary Table S3); based on the official KEGG BRITE classification, 23 pathways (24.0%) were immune-related—15 in the “Immune system” category (including Th17 cell differentiation, complement and coagulation cascades, hematopoietic cell lineage, and intestinal IgA production) and 8 classified as immune diseases (including rheumatoid arthritis, inflammatory bowel disease, allograft rejection, and graft-versus-host disease)—while 2 amino acid metabolism pathways (tryptophan metabolism, FDR = 0.015; phenylalanine metabolism, FDR = 0.041) were also significantly enriched, though not among the top-ranked pathways displayed (Fig. 2B; Supplementary Table S3). Consistently, GSEA of the full ranked gene list confirmed significant enrichment of immune-related GO biological process gene sets in the UCP2-high group, with adaptive immune response, lymphocyte-mediated immunity, and leukocyte-mediated immunity among the most prominently activated gene sets, while synaptic signalling and neurotransmitter-related gene sets were suppressed (Supplementary Fig. S4B; Supplementary Table S4).

### 3.5 PPI Network Analysis Identifies Immune Regulatory Hub Genes Co-Expressed with UCP2

**Figure 3:**
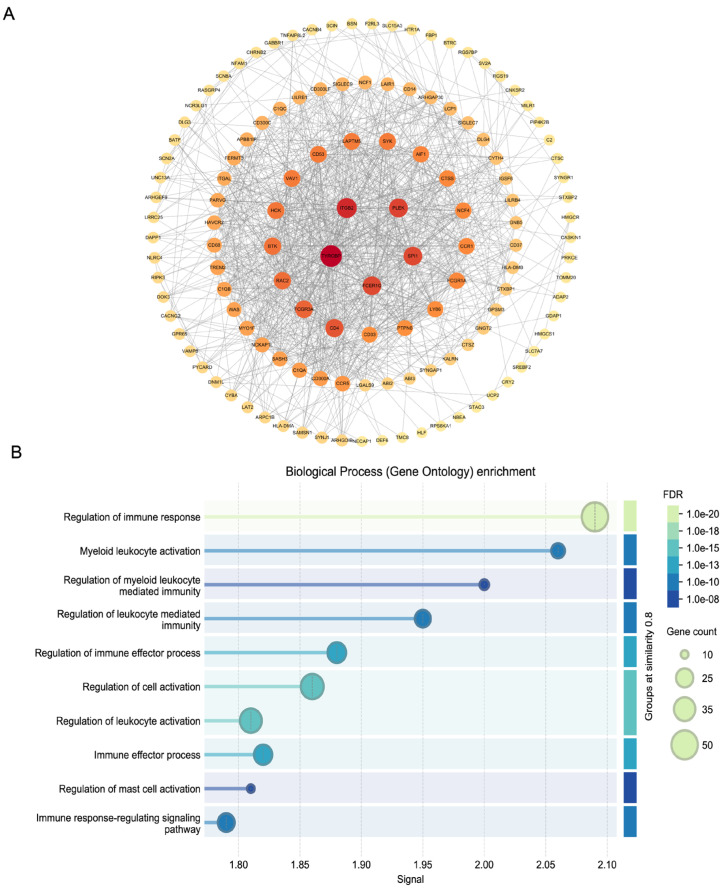
Protein–protein interaction network and functional enrichment analysis of DEGs associated with UCP2 high expression in glioma. (**A**) PPI network of DEGs between UCP2-high and UCP2-low groups, constructed using the STRING database (confidence score ≥ 0.4). Node size and colour intensity reflect degree of connectivity. UCP2 is indicated at the network periphery. (**B**) Bubble plot of the top ten most significantly enriched GO BP terms within the PPI network, as identified by STRING functional enrichment analysis. The *x*-axis represents the enrichment signal score; bubble size indicates gene count; bubble colour indicates statistical significance (FDR).

To characterise the protein-level interaction landscape associated with UCP2 high expression, the 1926 DEGs were submitted to STRING database analysis for protein–protein interaction (PPI) network construction. The resulting network comprised 191 nodes and 979 edges, with a mean node degree of 10.3 and a PPI enrichment *p* < 1.0 × 10^−^^16^, indicating that the network connectivity is significantly greater than expected by chance (Fig. 3A). The top ten hub genes ranked by degree of connectivity were TYROBP, SPI1, ITGB2, PLEK, FCER1G, BTK, VAV1, HCK, RAC2, and SYK, all of which occupied central positions within the network. The UCP2 node was located at the network periphery (Fig. 3A). Functional enrichment analysis of the PPI network demonstrated that constituent proteins were highly concentrated in immune-related biological processes; all top ten most significantly enriched pathways reached extreme significance (FDR < 1.0 × 10^−8^), with the immune response regulation pathway achieving an FDR of 1.0 × 10^−^^20^ (Fig. 3B).

### 3.6 UCP2 Expression Correlates with Lactate Metabolism-Related Genes and Macrophage Infiltration in Glioma

To explore the potential mechanistic basis linking UCP2 to tumour metabolic reprogramming and macrophage-driven immunosuppression, Spearman correlation analyses were performed across TCGA glioma samples (n = 670). UCP2 expression showed strong positive correlations with key lactate metabolism-related genes, with the strongest association observed for SLC16A3 (MCT4, *ρ* = 0.763, *p*.adj = 6.03 × 10^−^^128^), followed by HK2 (*ρ* = 0.593, *p*.adj = 2.00 × 10^−^^64^), LDHA (*ρ* = 0.426, *p*.adj = 1.28 × 10^−^^30^), and SLC16A1 (MCT1, *ρ* = 0.333, *p*.adj = 1.48 × 10^−^^18^). In contrast, LDHB—which preferentially catalyses the reverse conversion of lactate to pyruvate—was significantly negatively correlated with UCP2 (*ρ* = −0.295, *p*.adj = 8.80 × 10^−^^15^), consistent with a shift away from oxidative lactate utilisation in UCP2-high tumours. PKM2 showed a weak but statistically significant positive correlation (*ρ* = 0.090, *p*.adj = 0.023), whereas SLC2A1 (GLUT1) did not reach statistical significance (*ρ* = 0.055, *p*.adj = 0.157; Supplementary Fig. S5A). Collectively, the correlation pattern—strongly positive for lactate-producing enzymes and transporters, and negative for the lactate-consuming enzyme LDHB—suggests that UCP2-high tumours exhibit a metabolic profile favouring lactate production and export, consistent with the known role of lactate in promoting M2 macrophage polarisation. Consistent with the predominant macrophage/microglial expression pattern identified in the single-cell analyses, UCP2 expression was strongly positively correlated with CD68 mRNA levels across the same TCGA cohort (*ρ* = 0.862, *p* = 7.17 × 10^−^^199^; Supplementary Fig. S5B), providing bulk transcriptomic confirmation that the UCP2 signal in glioma tissue is largely attributable to macrophage/microglial infiltration. To further exclude the possibility that this association reflects differences in tumour cellularity rather than genuine macrophage infiltration, partial correlation analysis was performed controlling for ESTIMATE Score as a proxy for tumour purity (where higher ESTIMATE Score reflects greater stromal and immune cell infiltration rather than higher tumour cellularity). The UCP2–CD68 association remained highly significant after adjustment (partial *ρ* = 0.524, *p* = 1.80 × 10^−^^48^), confirming that the relationship between UCP2 expression and macrophage infiltration is not a spurious consequence of tumour purity variation (Supplementary Fig. S5C).

### 3.7 UCP2-High Expression Is Associated with an Immunosuppressive Tumour Microenvironment and Predicted Resistance to Immunotherapy

**Figure 4:**
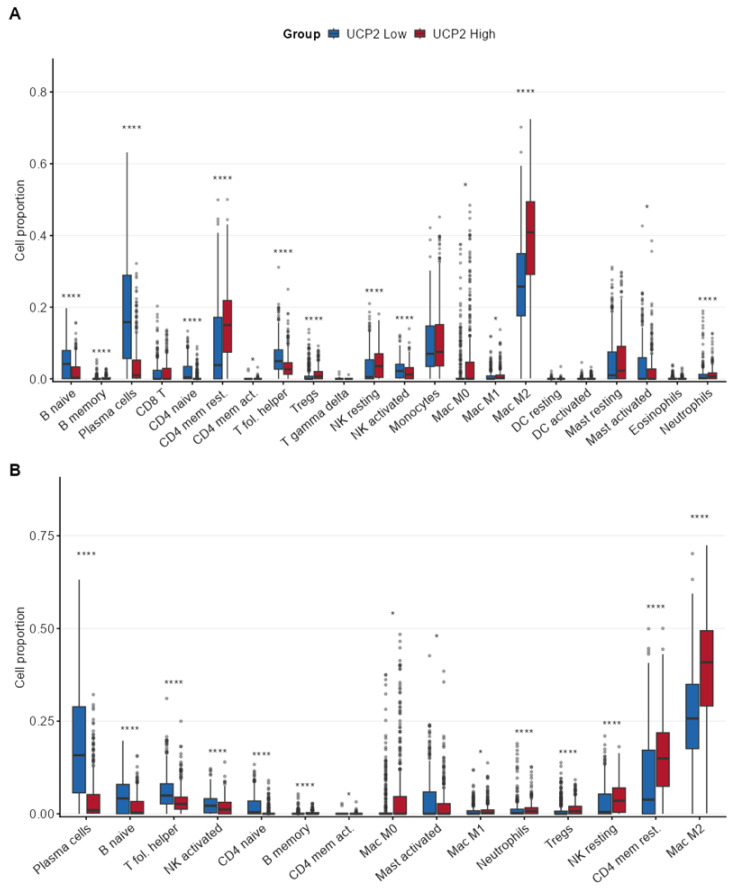
UCP2 high expression is associated with remodelling of the tumour immune microenvironment in glioma. (**A**) Boxplots showing the estimated proportions of 22 immune cell subtypes by CIBERSORT (LM22) in UCP2-high and UCP2-low glioma patients (TCGA cohort, n = 670). Blue, UCP2-low; red, UCP2-high. Significance markers indicate Wilcoxon rank-sum test results (**B**) Enlarged boxplots of the 15 immune cell subtypes with statistically significant differences between groups (*p* < 0.05), ordered by effect size (median difference, high minus low) from left to right. *p < 0.05, ****p < 0.0001.

CIBERSORT deconvolution of 670 TCGA glioma samples revealed significant differences in 15 of 22 immune cell subtypes between UCP2-high and UCP2-low groups (Wilcoxon rank-sum test, Benjamini–Hochberg adjusted *p* < 0.05; Fig. 4A; Supplementary Table S5). The significantly differentially infiltrated immune cell subtypes are shown in Fig. 4B.

In UCP2-high tumours, five cell populations were significantly enriched. M2 macrophages showed the most pronounced enrichment (median 0.41 vs. 0.26, *p*.adj = 1.18 × 10^−^^26^), followed by resting CD4^+^ memory T cells (median 0.150 vs. 0.038, *p*.adj = 3.01 × 10^−^^14^), regulatory T cells (Tregs, median 0.007 vs. 0, *p*.adj = 6.78 × 10^−^^15^), resting natural killer (NK) cells (median 0.035 vs. 0.005, *p*.adj = 6.08 × 10^−^^8^), and neutrophils (median 0.007 vs. 0.002, *p*.adj = 3.99 × 10^−^^6^). M1 macrophages were also modestly but significantly enriched in UCP2-high tumours (median 0.0022 vs. 0.0002, *p*.adj = 2.49 × 10^−^^2^); however, the magnitude of M2 enrichment was substantially greater, consistent with a net immunosuppressive macrophage polarisation state.

Conversely, nine immune cell populations were significantly depleted in UCP2-high tumours, including plasma cells (median 0.010 vs. 0.158, *p*.adj = 2.27 × 10^−^^41^), naive CD4^+^ T cells (median 0 vs. 0.004, *p*.adj = 2.11 × 10^−^^22^), follicular helper T cells (median 0.026 vs. 0.049, *p*.adj = 6.79 × 10^−^^18^), naive B cells (median 0.003 vs. 0.042, *p*.adj = 1.50 × 10^−^^11^), memory B cells (*p*.adj = 4.74 × 10^−^^9^), activated NK cells (median 0.012 vs. 0.022, *p*.adj = 7.27 × 10^−^^5^), M0 macrophages (*p*.adj = 2.01 × 10^−^^2^), activated CD4^+^ memory T cells (*p*.adj = 2.09 × 10^−^^2^), and activated mast cells (*p*.adj = 4.05 × 10^−^^2^).

**Figure 5:**
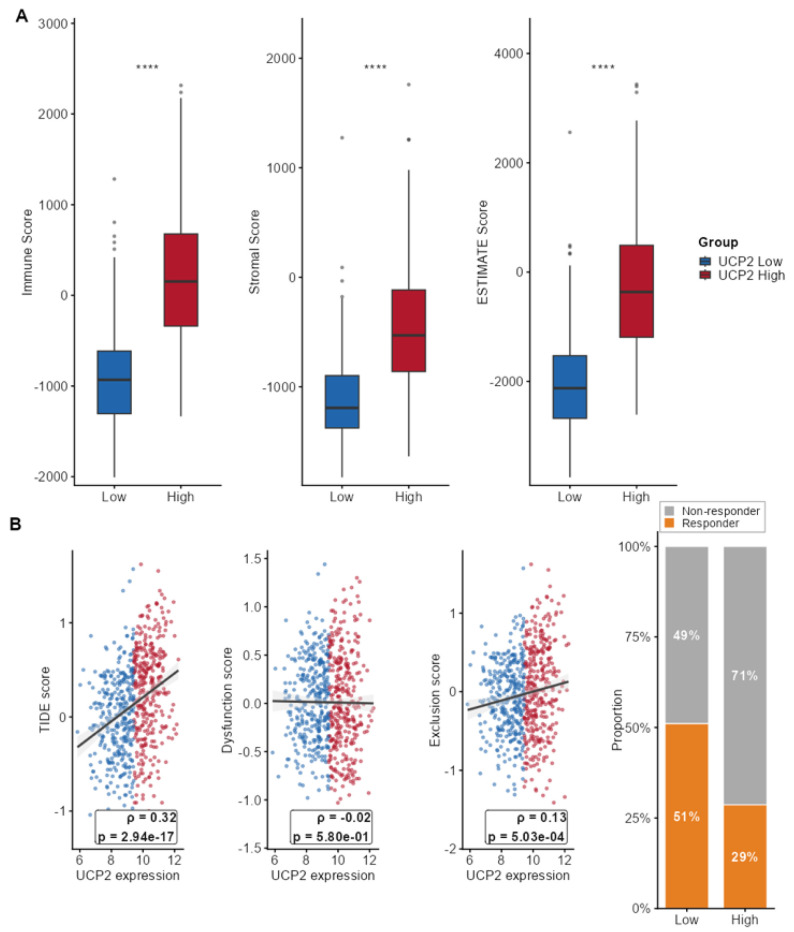
UCP2 expression is associated with an immunosuppressive tumour microenvironment and predicted resistance to immunotherapy in glioma. (**A**) Boxplots comparing ESTIMATE immune, stromal, and ESTIMATE scores between UCP2-high and UCP2-low groups (TCGA cohort, n = 670). Blue, UCP2-low; red, UCP2-high. Significance assessed by Wilcoxon rank-sum test (****p < 0.0001). (**B**) Spearman correlations between UCP2 expression and TIDE score, T cell dysfunction score, and T cell exclusion score (left three panels). Each point represents one tumour sample; regression lines with 95% confidence intervals are shown. Proportions of predicted immunotherapy responders and non-responders by TIDE algorithm in UCP2-high and UCP2-low groups (right panel); *p* value by Fisher’s exact test.

Collectively, these findings indicate that UCP2-high tumours harbour an immunosuppressive immune landscape characterised by pronounced myeloid and regulatory cell enrichment alongside broad depletion of effector and humoral immune populations. Consistent with this immunosuppressive profile, ESTIMATE analysis demonstrated that all three scores were significantly elevated in the UCP2-high group compared with the UCP2-low group, including Immune Score (*p* < 0.0001), Stromal Score (*p* < 0.0001), and ESTIMATE Score (*p* < 0.0001), indicating a substantially remodelled tumour microenvironment with higher immune and stromal infiltration (Fig. 5A).

To assess the potential implications of UCP2 expression for immunotherapy response, TIDE analysis was performed. UCP2 expression showed a significant positive correlation with the overall TIDE score (ρ = 0.32, *p* = 2.94 × 10^−^^17^; Fig. 5B). Decomposition of the two core TIDE components revealed that UCP2 was significantly positively correlated with the Exclusion score (ρ = 0.13, *p* = 5.03 × 10^−^^4^), whereas no significant correlation was observed with the Dysfunction score (ρ = −0.02, *p* = 0.580), suggesting that the immunosuppressive phenotype associated with UCP2 is predominantly reflected in the Exclusion dimension of TIDE; the absence of a significant Dysfunction score correlation does not, however, exclude T cell exhaustion as a contributing mechanism. The proportion of predicted non-responders was significantly higher in the UCP2-high group (71%) compared with the UCP2-low group (49%; Fisher’s exact test, *p* = 4.41 × 10^−^^9^; Fig. 5B). Furthermore, within the predicted non-responder subgroup (n = 403), UCP2 expression remained significantly positively correlated with TIDE score (ρ = 0.321, *p* = 3.92 × 10^−^^11^), suggesting that UCP2 may carry additional stratification value within this clinically challenging population.

### 3.8 UCP2 Expression Is Strongly Correlated with Immune Checkpoints, Particularly HAVCR2, across Bulk and Single-Cell Transcriptomic Data

**Figure 6:**
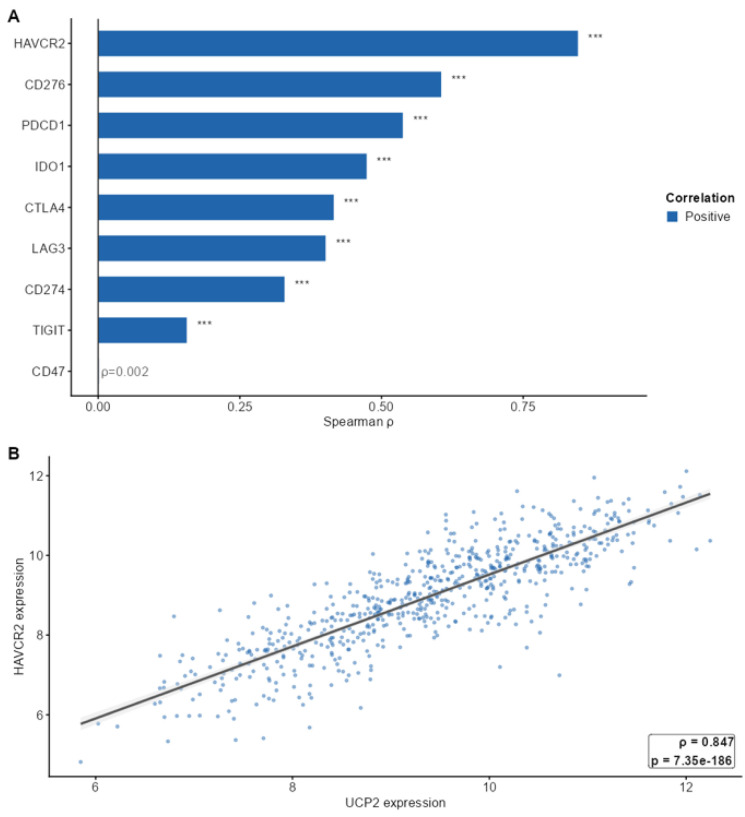
UCP2 expression is positively correlated with immune checkpoint gene expression in glioma. (**A**) Bar plot showing Spearman correlation coefficients (ρ) between UCP2 and nine immune checkpoint genes (TCGA cohort, n = 670). Significance markers indicate Benjamini–Hochberg-adjusted *p* values (***FDR < 0.001). (**B**) Scatter plot of UCP2 versus HAVCR2 expression. Each point represents one tumour sample; regression line with 95% confidence interval is shown.

Among nine immune checkpoint genes examined in the TCGA glioma cohort, UCP2 expression showed the strongest positive correlation with HAVCR2 (TIM-3, ρ = 0.847, *p* < 0.001), followed by CD276 (B7-H3, ρ = 0.606, *p* < 0.001), PDCD1 (PD-1, ρ = 0.538, *p* < 0.001), IDO1 (ρ = 0.475, *p* < 0.001), CTLA4 (ρ = 0.417, *p* < 0.001), LAG3 (ρ = 0.402, *p* < 0.001), CD274 (PD-L1, ρ = 0.330, *p* < 0.001), and TIGIT (ρ = 0.157, *p* < 0.001), while CD47 showed no significant correlation (ρ = 0.002, *p* = 0.951; Fig. 6A,B). The exceptionally strong correlation with HAVCR2 suggests a particularly close functional relationship between UCP2 and TIM-3-mediated immune suppression in the glioma microenvironment. To determine whether this association reflects genuine co-expression within the same cell population rather than a spurious bulk-level correlation, UCP2 and HAVCR2 co-expression was examined specifically within macrophage/microglial clusters across the three independent scRNA-seq datasets (total macrophage/microglial cells: n = 3074). Spearman correlation analysis confirmed a significant positive correlation between UCP2 and HAVCR2 in GSE84465 (ρ = 0.189, *p* = 1.27 × 10^−^^15^, n = 1756) and GSE89567 (ρ = 0.088, *p* = 0.004, n = 1083), while the correlation in GSE70630 did not reach statistical significance (ρ = 0.112, *p* = 0.086, n = 235), likely attributable to the smaller macrophage/microglial cell count in this dataset. In the combined macrophage/microglial population across all three datasets, a significant positive correlation was observed (ρ = 0.268, *p* = 7.15 × 10^−^^52^, n = 3074). These findings confirm that UCP2 and HAVCR2 are co-expressed at the single-cell level within tumour-associated macrophage/microglial populations, providing cell-type-resolved evidence for their functional association in the glioma immune microenvironment.

### 3.9 UCP2 Expression Progressively Increases along the TAM Immunosuppressive Trajectory

To investigate whether UCP2 expression is associated with TAM functional state transitions in the glioma microenvironment, pseudotime trajectory analysis was performed on the pooled macrophage/microglial population of 3074 TAMs derived from the three independent scRNA-seq cohorts (GSE70630, n = 235; GSE84465, n = 1756; GSE89567, n = 1083). Following Seurat-based re-processing and UMAP embedding of the merged TAM population, Slingshot inferred multiple differentiation trajectories spanning UMAP space. The principal lineage most strongly correlated with UCP2 expression was selected for downstream analysis, comprising 1575 TAMs with finite pseudotime assignments (GSE70630, n = 60; GSE84465, n = 795; GSE89567, n = 720) and spanning a pseudotime range of 0 to 25.75. UMAP visualisation confirmed that pseudotime progression and UCP2 expression followed a consistent spatial pattern across all three datasets (Supplementary Fig. S6A).

UCP2 expression showed a statistically significant positive correlation with pseudotime progression (Spearman ρ = 0.217, *p* = 2.89 × 10^−^^18^), indicating that UCP2 levels increase as TAMs advance along the inferred trajectory (Supplementary Fig. S6B). Consistent with a progressive shift toward an immunosuppressive phenotype, several canonical M2 and exhaustion markers were also positively correlated with pseudotime, including AIF1 (ρ = 0.615, *p* = 1.33 × 10^−^^164^), IL10 (ρ = 0.478, *p* = 1.07 × 10^−^^90^), TGFB1 (ρ = 0.302, *p* = 1.56 × 10^−^^34^), HAVCR2 (ρ = 0.282, *p* = 3.31 × 10^−^^30^), MRC1 (ρ = 0.213, *p* = 1.27 × 10^−^^17^), and CD163 (ρ = 0.077, *p* = 0.002) (Supplementary Fig. S6C). Although pro-inflammatory markers including TNF (ρ = 0.198, *p* = 2.25 × 10^−^^15^) and CD86 (ρ = 0.148, *p* = 3.26 × 10^−^^9^) also increased along pseudotime, module-level analysis revealed a divergent polarisation pattern: the M2 immunosuppressive module score significantly increased with pseudotime (ρ = 0.149, *p* = 3.09 × 10^−^^9^), whereas the M1 pro-inflammatory module score exhibited a modest but significant negative correlation (ρ = −0.065, *p* = 0.00987) (Supplementary Fig. S6D). Collectively, these findings demonstrate that UCP2 upregulation co-occurs with the progressive acquisition of an immunosuppressive TAM state, suggesting a potential role for UCP2 in driving or sustaining TAM immunosuppressive polarisation in the glioma microenvironment.

### 3.10 UCP2 Expression Is an Independent Prognostic Factor in Glioma

**Figure 7:**
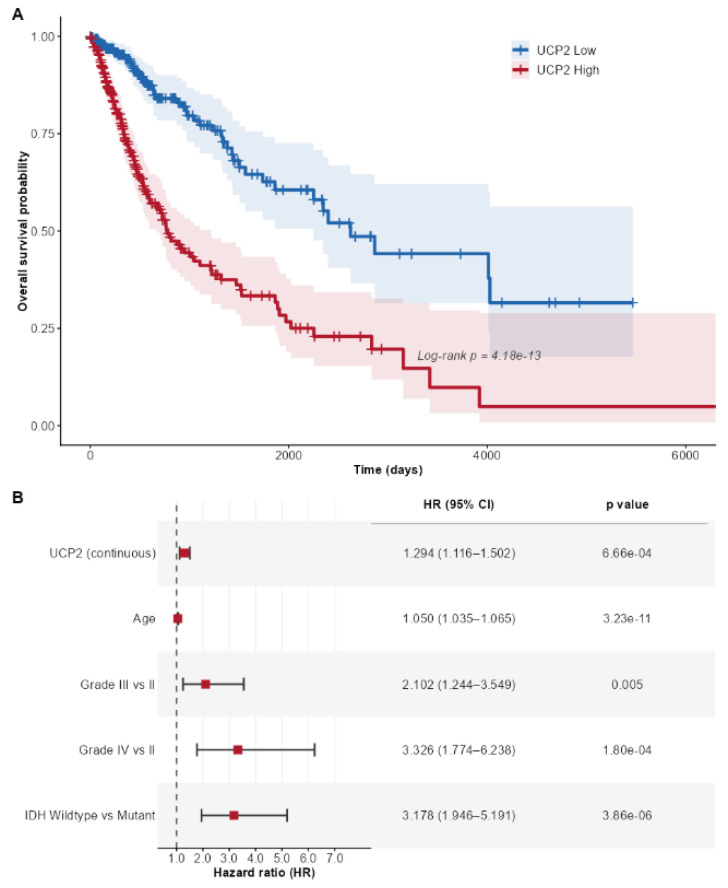
UCP2 expression is an independent prognostic factor in glioma. (**A**) Kaplan–Meier overall survival curves for UCP2-high (**red**) and UCP2-low (**blue**) patient groups (TCGA cohort, n = 597), stratified by median UCP2 expression. Shaded areas represent 95% confidence intervals; tick marks indicate censored observations. *p* value by log-rank test. (**B**) Forest plot of multivariate Cox proportional hazards regression incorporating UCP2 expression, age, WHO grade, and IDH status. Filled squares indicate hazard ratio point estimates; horizontal lines represent 95% confidence intervals. Red squares indicate covariates reaching statistical significance (*p* < 0.05). HR, hazard ratio; CI, confidence interval.

To evaluate the prognostic significance of UCP2 expression, survival analysis was performed in the TCGA glioma cohort (n = 670; n = 597 with available survival data). Kaplan–Meier analysis demonstrated that UCP2-high patients exhibited significantly worse overall survival compared to UCP2-low patients (log-rank test, *p* = 4.18 × 10^−^^13^; Fig. 7A). Univariate Cox regression confirmed that elevated UCP2 expression was significantly associated with poor prognosis (HR = 1.604, 95% CI: 1.405–1.832, *p* = 2.95 × 10^−^^12^). In multivariate Cox regression incorporating age, WHO grade, and IDH mutation status, UCP2 remained an independent prognostic factor (HR = 1.294, 95% CI: 1.116–1.502, *p* = 6.66 × 10^−^^4^; Fig. 7B). Among the covariates, age was independently associated with survival (HR = 1.050, 95% CI: 1.035–1.065, *p* = 3.23 × 10^−^^11^). Compared with WHO Grade II (reference), both Grade III (HR = 2.102, 95% CI: 1.244–3.549, *p* = 0.005) and Grade IV (HR = 3.326, 95% CI: 1.774–6.238, *p* = 1.80 × 10^−^^4^) were independently associated with worse survival. IDH wildtype status was also independently associated with poor prognosis (HR = 3.178, 95% CI: 1.946–5.191, *p* = 3.86 × 10^−^^6^; Fig. 7B). Survival data were not available for the institutional IHC cohort (n = 96); prognostic validation in an independent clinical cohort with follow-up data represents an important direction for future work.

### 3.11 IHC Validation in Clinical Glioma Samples

**Figure 8:**
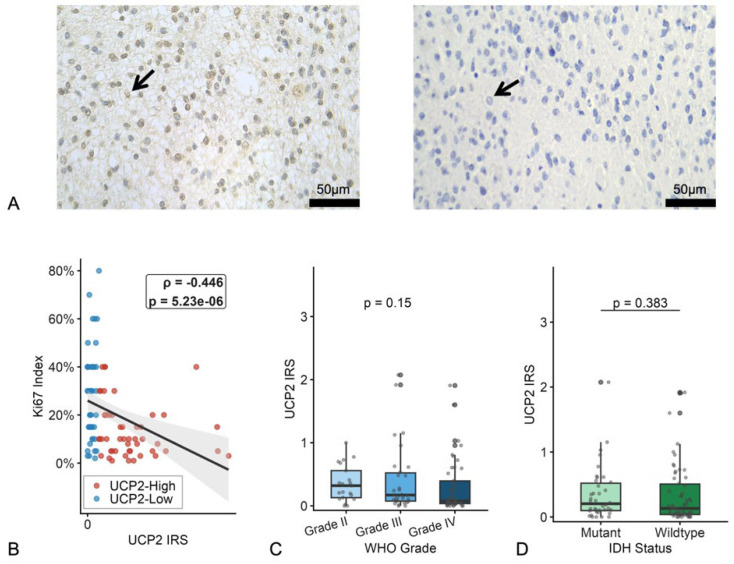
UCP2 protein expression in clinical glioma samples and its correlation with clinicopathological features. (**A**) Representative IHC images of UCP2 staining in glioma tissue: high expression with strong cytoplasmic staining (**left**) and negative staining (**right**). Black arrows indicate representative cells. Scale bar = 50 μm. (**B**) Scatter plot of UCP2 IRS versus Ki67 proliferation index (n = 96). Points are coloured by expression group (red, UCP2-high; blue, UCP2-low); regression line with 95% confidence interval is shown. Spearman correlation coefficient and *p* value are displayed within the panel. (**C**) Boxplots of UCP2 IRS across WHO tumour grades (Kruskal–Wallis test). (**D**) Boxplots of UCP2 IRS by IDH mutation status (Wilcoxon rank-sum test). Individual data points are overlaid.

Representative IHC images demonstrated cytoplasmic UCP2 protein expression in glioma tissue, with clear contrast between UCP2-positive and UCP2-negative cases (Fig. 8A). Patients were stratified into UCP2-high (n = 48) and UCP2-low (n = 48) groups using the median immunoreactivity score (IRS) as the grouping cutoff. The two groups were comparable in age, sex, WHO grade, IDH status, and p53 status (all *p* > 0.05, Table 1). UCP2 protein expression was significantly negatively correlated with Ki67 proliferation index (Spearman ρ = −0.446, *p* = 5.23 × 10^−^^6^; Fig. 8B). UCP2-high tumours showed markedly lower Ki67 values compared to UCP2-low tumours (median 0.10 vs. 0.30, *p* = 1.68 × 10^−^^5^; Table 1). No significant associations were observed between UCP2 protein expression and WHO grade (*p* = 0.059, Table 1) or IDH status (*p* = 0.531, Table 1), findings further corroborated by direct comparison of UCP2 IRS across WHO grade subgroups and IDH status groups (*p* = 0.15 and *p* = 0.383, respectively; Fig. 8C,D).

**Table 1:** Clinicopathological characteristics of glioma patients stratified by UCP2 protein expression (n = 96).

Variable	UCP2-High (n = 48)	UCP2-Low (n = 48)	*p* Value
Age (years), median (IQR)	52 (44–61)	52 (43–61)	0.834
Sex, n (%)	-	-	0.218
Male	30 (62.5%)	23 (47.9%)	-
Female	18 (37.5%)	25 (52.1%)	-
WHO Grade, n (%)	-	-	0.059
Grade II	15 (31.2%)	6 (12.5%)	-
Grade III	15 (31.2%)	15 (31.2%)	-
Grade IV	18 (37.5%)	27 (56.2%)	-
IDH Status, n (%)	-	-	0.531
Mutant	21 (43.8%)	17 (35.4%)	-
Wildtype	27 (56.2%)	31 (64.6%)	-
p53 Status, n (%)	-	-	0.116
Positive	1 (2.1%)	6 (12.5%)	-
Negative	47 (97.9%)	42 (87.5%)	-
Ki67 Index, median (IQR)	0.10 (0.05–0.20)	0.30 (0.15–0.40)	<0.001

Note: Continuous variables are expressed as median (interquartile range, IQR); categorical variables are expressed as frequency (percentage). The Mann–Whitney U test was used for continuous variables; the chi-square test was used for categorical variables. Bold *p* value indicates statistical significance (*p* < 0.05). IRS, immunoreactive score; WHO, World Health Organization; IDH, isocitrate dehydrogenase.

### 3.12 UCP2 Protein Co-Localises with CD68^+^ Macrophages in Glioma Tissue

**Figure 9:**
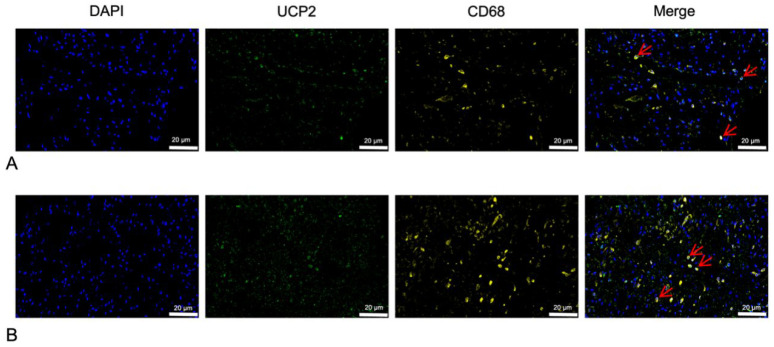
Immunofluorescence co-staining of UCP2 and CD68 in representative glioma specimens. (**A**) WHO Grade IV glioblastoma (IDH wildtype). (**B**) WHO Grade III glioma (IDH wildtype). Each row displays DAPI (blue), UCP2 (green), CD68 (yellow), and merged composite image (left to right). Red arrows indicate UCP2^+^CD68^+^ double-positive cells. Scale bar = 20 μm.

To confirm the spatial co-localisation of UCP2 protein with tumour-associated macrophages at the tissue level, immunofluorescence dual-labelling for UCP2 (green, CoraLite Plus 488) and the pan-macrophage marker CD68 (yellow, CoraLite Plus 555) was performed on FFPE sections from six glioma specimens drawn from the clinical cohort. Co-localisation of UCP2 and CD68 signals was identified in two representative cases (Fig. 9A,B): a 53-year-old male with WHO Grade IV glioblastoma (IDH wildtype) and a 48-year-old male with WHO Grade III glioma (IDH wildtype). In merged fluorescence images, UCP2^+^CD68^+^ double-positive cells—indicated by red arrows—produced overlapping signals, confirming the presence of UCP2-expressing macrophages within the tumour parenchyma. These findings provide protein-level spatial evidence corroborating the predominant macrophage/microglial localisation of UCP2 identified in single-cell RNA sequencing analyses, and support the interpretation that UCP2 immunostaining in glioma tissue primarily reflects TAM infiltration rather than tumour cell-intrinsic expression.

## 4 Discussion

Although UCP2 has been extensively studied as a metabolic regulator in glioma—with established roles in aerobic glycolysis, ROS attenuation, and therapy resistance—its relationship with the glioma immune microenvironment has remained largely unexplored. Vallejo et al. [9] proposed that UCP2 upregulation may dampen macrophage immune responses by limiting ROS generation and suggested that this immunosuppressive effect warrants further investigation in the tumor context. Luby and Alves-Guerra further noted that despite growing evidence linking UCP2 to antitumor immunity, this dimension of UCP2 biology remains little studied across cancer types [10]. Furthermore, Guo et al. [12] conducted a comprehensive pan-cancer analysis confirming UCP2 as a prognostic indicator and linking it to immune-related pathways in glioma using bulk multi-omics data; however, bulk transcriptomic approaches inherently lack the cellular resolution necessary to identify which specific subpopulations drive UCP2 overexpression within the heterogeneous TME. Notably, their pan-cancer findings of negative associations between UCP2 and immunosuppressive cell infiltration may reflect tumor-type-specific differences, as glioma harbours a uniquely macrophage-dominated immunosuppressive microenvironment that may not be captured at the pan-cancer level. The present study directly addresses these limitations by integrating multi-omics approaches—including bulk transcriptomics, single-cell RNA sequencing, and protein-level validation—consistent with the emerging multi-omics paradigm in cancer biomarker discovery [39] to provide single-cell resolution of UCP2 expression across three independent glioma datasets. Across all three datasets, UCP2 expression within the glioma TME was consistently and predominantly enriched in macrophage/microglial populations relative to tumor cells—a finding that suggests a reframing of UCP2 from a tumor cell-autonomous oncogene to a potential marker of TAM-associated immunosuppression and provides a transcriptomic basis for the immunosuppressive associations observed in prior bulk analyses.

Consistent with previous reports [9,12], pan-cancer analysis confirmed broad UCP2 upregulation across human malignancies, with glioma showing the most pronounced overexpression relative to normal brain tissue (Supplementary Figs. S1 and S2). However, bulk transcriptomic data alone cannot resolve the cellular origin of this signal within the heterogeneous glioma TME. Single-cell analysis of three independent datasets consistently indicated that UCP2 expression was predominantly enriched in macrophage/microglial populations rather than tumor cells or other major cell types (Fig. 1), suggesting that TAM infiltration rather than tumor cell-intrinsic upregulation may be the primary contributor to elevated UCP2 levels in glioma bulk transcriptomes.

If UCP2 overexpression in glioma reflects TAM infiltration rather than tumour cell-intrinsic upregulation, the transcriptomic signature of UCP2-high tumours would be expected to be dominated by immune rather than metabolic gene programmes. Functional enrichment analysis confirmed this prediction: the majority of significantly enriched GO biological process terms and KEGG pathways were immune-related, encompassing adaptive immunity, myeloid cell differentiation, cytokine production, and complement activation—hallmarks of macrophage/microglial transcriptional activity—whereas metabolic pathways represented only a minor fraction (Fig. 2; Supplementary Tables S2 and S3). This immunological dominance stands in contrast to what would be expected if UCP2 were primarily expressed by tumour cells, where metabolic reprogramming pathways would be predicted to predominate, as demonstrated in UCP2-overexpressing glioma cell lines via p38 MAPK signalling [11]. GSEA further reinforced this pattern, with adaptive immune response as the most strongly enriched gene set, while negatively enriched pathways were predominantly neuronal—consistent with relative depletion of neuronal programmes in tumours with greater immune infiltration (Supplementary Fig. S4B). Corroborating these findings at the protein interaction level, PPI network analysis identified hub genes including TYROBP, SPI1, ITGB2, PLEK, and FCER1G—all established immune cell signalling and leukocyte activation proteins—with network connectivity showing extreme enrichment for immune regulatory pathways (Fig. 3). Notably, UCP2 itself occupied a peripheral position within this network, further suggesting that UCP2 in glioma represents a marker of TAM infiltration rather than a central driver of tumour cell proliferation.

To further substantiate the TAM-intrinsic nature of UCP2 at the bulk transcriptomic level, UCP2 expression was examined in relation to macrophage infiltration markers and tumour purity across the TCGA glioma cohort. UCP2 showed a strong positive correlation with CD68 mRNA, and this association remained highly significant after controlling for ESTIMATE Score as a proxy for tumour purity (Supplementary Fig. S5B,C), confirming that the UCP2–macrophage relationship reflects genuine immune infiltration rather than a spurious consequence of variation in tumour cellularity—directly addressing a key methodological concern in tumour immune profiling [34].

Complementing these findings, UCP2 expression showed strong positive correlations with key lactate-producing genes including SLC16A3 (MCT4), HK2, LDHA, and SLC16A1 (MCT1), while showing a significant negative correlation with LDHB—an enzyme that preferentially converts lactate back to pyruvate (Supplementary Fig. S5A). This bidirectional pattern suggests that UCP2-high tumours operate in a metabolic state favouring lactate production and export. This observation is mechanistically significant: tumour-derived lactate has been shown to promote immunosuppressive M2 polarisation of macrophages through multiple mechanisms including histone H3K18 lactylation as a key epigenetic mechanism—a process specifically demonstrated in glioblastoma via the MCT1/H3K18La/TNFSF9 axis [40]. Together, these correlations provide transcriptomic-level support for a UCP2–lactate–TAM polarisation axis, whereby UCP2-associated metabolic reprogramming may contribute to the immunosuppressive remodelling of the glioma TME [40]. 

Consistent with this proposed axis, CIBERSORT deconvolution revealed a markedly immunosuppressive immune landscape in UCP2-high tumours, characterised by pronounced enrichment of M2 macrophages and regulatory T cells alongside broad depletion of effector and humoral immune populations (Fig. 4; Supplementary Table S5). ESTIMATE analysis further demonstrated significantly elevated Immune Score, Stromal Score, and ESTIMATE Score in UCP2-high tumours (Fig. 5A), reflecting a ‘recruitment-suppression’ paradox: high overall immune infiltration dominated by immunosuppressive rather than effector populations. M2-polarised TAMs within this landscape are known to secrete interleukin-10 (IL-10) and transforming growth factor-β (TGF-β), cooperate with Tregs to construct an immunosuppressive network, and upregulate immune checkpoint molecules [41]—collectively creating a microenvironment hostile to anti-tumour immunity.

The immunosuppressive landscape associated with UCP2 high expression was further reflected in strong positive correlations with multiple immune checkpoint molecules, most prominently HAVCR2/TIM-3, followed by CD276, PDCD1, IDO1, CTLA4, LAG3, and CD274 (Fig. 6). The particularly strong association with TIM-3 is mechanistically coherent: TIM-3 functions not only as a canonical marker of T cell exhaustion [42] but also as an active regulator of M2 macrophage polarisation, suppressing innate anti-tumour inflammatory responses through the signal transducer and activator of transcription 1 (STAT1)-miR-155-suppressor of cytokine signalling 1 (SOCS1) signalling axis [43]—making it the checkpoint most directly reflective of the M2 TAM-dominated immunosuppressive phenotype associated with UCP2.

To determine whether this association reflects genuine co-expression within the same TAM populations rather than a population-level confound, UCP2 and HAVCR2 co-expression was examined specifically within macrophage/microglial clusters across the three independent scRNA-seq datasets. A consistent and significant positive correlation was observed in the combined macrophage/microglial population (Fig. 6), confirming that the exceptionally strong bulk-level correlation reflects genuine co-expression within individual TAMs. This finding suggests that UCP2-high macrophages may represent a functionally distinct, deeply immunosuppressive subpopulation within the glioma TME—one in which TIM-3-mediated suppression of innate immune activation operates in concert with UCP2-associated metabolic reprogramming.

To determine whether UCP2 expression is dynamically linked to TAM immunosuppressive polarisation rather than simply co-expressed in a static population, pseudotime trajectory analysis was performed using the Slingshot algorithm on the pooled TAM population across all three independent scRNA-seq datasets. UCP2 expression showed a significant positive correlation with pseudotime progression along the principal immunosuppressive lineage (Supplementary Fig. S6B), indicating that UCP2 levels increase progressively as TAMs advance toward a more immunosuppressive functional state. This trajectory was accompanied by the coordinated upregulation of canonical M2 and exhaustion markers—including AIF1, IL10, TGFB1, HAVCR2, MRC1, and CD163—alongside a divergent module-level pattern in which the M2 immunosuppressive module score increased while the M1 pro-inflammatory module score exhibited a modest but significant negative trend (Supplementary Fig. S6C,D). Although individual pro-inflammatory markers such as TNF and CD86 also showed positive correlations with pseudotime—consistent with the known transcriptional complexity and heterogeneity of TAM states in glioma [44]—the divergent module-level pattern indicates a net shift toward immunosuppression rather than a generalised activation state. Together, these findings suggest that UCP2 upregulation is not merely a static correlate of TAM abundance but a dynamic feature of TAM immunosuppressive polarisation, rising progressively as TAMs transition toward a functionally suppressive phenotype. This temporal co-occurrence positions UCP2 as a potential participant in—rather than a passive bystander of—the immunosuppressive remodelling of the glioma TME.

TIDE analysis provided further insight into the immunotherapy resistance profile associated with UCP2 expression. Decomposition of the two core TIDE components revealed that UCP2 was significantly positively correlated with the Exclusion score but not with the Dysfunction score (Fig. 5B), suggesting that the immune resistance associated with UCP2-high expression may predominantly operate through suppression of T cell infiltration into the tumour microenvironment. This pattern is consistent with the CIBERSORT and ESTIMATE findings described above, which collectively indicate that UCP2-high tumours harbour a deeply immunosuppressive microenvironment characterised by M2 TAM and Treg enrichment—cell populations known to actively exclude effector T cells from tumour parenchyma [41]. However, given the correlative nature of these analyses, the absence of a significant Dysfunction score correlation does not exclude T cell exhaustion as a contributor, and functional validation will be required to fully characterise the mechanistic basis of immune resistance. Notably, UCP2 expression remained significantly correlated with overall TIDE score within the predicted non-responder subgroup (Fig. 5B), suggesting that UCP2 may carry additional stratification value within this clinically challenging population.

The biological significance of the UCP2–TAM immunosuppressive axis is further underscored by its independent prognostic value. Kaplan–Meier analysis demonstrated significantly worse overall survival in UCP2-high patients, and multivariate Cox regression confirmed that UCP2 remained an independent prognostic factor after adjustment for age, WHO grade, and IDH mutation status (Fig. 7). Notably, the prognostic effect of UCP2 persisted after controlling for IDH wildtype status and WHO Grade IV—two of the strongest known adverse prognostic factors in glioma—indicating that UCP2 captures prognostic information not fully explained by established clinicopathological variables.

Current prognostic stratification of glioma relies heavily on WHO grade and IDH status [45], yet within each molecular subgroup there remains substantial outcome heterogeneity incompletely explained by these variables alone. UCP2 expression may capture a component of this residual heterogeneity by reflecting the immunosuppressive state of the TME—a dimension increasingly recognised as a determinant of both natural disease course and therapeutic response in glioma [46]. The convergence of independent prognostic significance with immunosuppressive TME features and predicted immunotherapy resistance suggests that UCP2-high tumours may represent a clinically distinct subgroup characterised by TAM-driven immunosuppression, for whom combination strategies targeting TAM repolarisation or metabolic reprogramming may warrant further investigation.

To contextualise these *in vivo* findings with respect to prior *in vitro* evidence, UCP2 expression was examined across 74 unique glioma cell lines in the CCLE dataset. UCP2 was detectably expressed in 73 of 74 cell lines (median = 4.09 log_2_(TPM + 1); Supplementary Fig. S3), confirming that glioma tumour cells possess intrinsic UCP2 expression capacity in the absence of any microenvironmental component. Finally, the inverse correlation between UCP2 protein expression and Ki67 proliferation index observed in clinical samples (Fig. 8B) appears paradoxical given *in vitro* evidence that UCP2 promotes glioblastoma cell proliferation via the p38 MAPK pathway [11]. However, this apparent paradox is readily explained by the scRNA-seq finding that UCP2 expression in the glioma TME is predominantly localised to macrophage/microglial rather than tumour cell populations: high UCP2 immunostaining in tissue sections (Fig. 8A) is therefore more likely to reflect TAM infiltration than tumour cell abundance. Consistent with this interpretation, TAM-rich tumour regions would be expected to exhibit lower Ki67 indices, as Ki67 expression is restricted to actively cycling cells and is absent in post-mitotic or resting cells including macrophages [47].

The absence of a significant association between UCP2 protein levels and WHO grade or IDH status in our clinical cohort (Fig. 8C,D) further supports this interpretation. TAM infiltration occurs across all glioma grades and is not restricted to high-grade tumours: although IDH-wildtype GBMs generally harbour greater numbers of bone marrow-derived macrophages, IDH-mutant lower-grade gliomas contain substantial microglia-derived TAM populations [44]. This cellular basis was directly confirmed at the protein level by immunofluorescence co-staining, in which UCP2^+^CD68^+^ double-positive cells were identified within the tumour parenchyma across representative cases (Fig. 9A,B). Collectively, these findings suggest that UCP2 immunostaining in glioma tissue primarily reflects the immunosuppressive state of the TAM-dominated TME rather than intrinsic tumour cell characteristics, and that UCP2 may serve as a grade- and IDH-independent indicator of immune microenvironmental composition.

## 5 Limitations

Several limitations of the present study should be acknowledged. First, all transcriptomic analyses are based on publicly available datasets and are inherently correlative in nature; causal relationships between UCP2 expression, TAM polarisation, and immunosuppressive remodelling of the glioma TME remain to be established through functional experiments. Second, although immunofluorescence co-staining for UCP2 and CD68 confirmed co-localisation at the tissue level, this validation was conducted on a limited number of FFPE sections (n = 6), restricting the generalisability of the finding. Larger-scale spatial validation incorporating quantitative co-localisation analysis across the full clinical cohort is warranted in future studies. Third, the IHC validation cohort comprised 96 patients from a single institution using a retrospective cross-sectional design, which limits generalisability and introduces potential selection bias. A formal sample size justification was not performed prior to cohort assembly, and the relatively modest cohort size may have limited statistical power to detect associations with WHO grade and IDH status at the protein level. Furthermore, overall survival data were not available for the institutional IHC cohort, as this was a retrospective cross-sectional study using archival tissue specimens without linked follow-up records; independent prognostic validation in a clinical cohort with prospective survival data represents an important direction for future work. Fourth, although partial correlation analysis confirmed that the UCP2–CD68 association remained significant after controlling for ESTIMATE Score, the possibility that residual tumour purity confounding influences other immune signature analyses—including CIBERSORT deconvolution and immune checkpoint correlations—cannot be entirely excluded. Future studies incorporating single-nucleus RNA sequencing or spatial transcriptomics would provide a more definitive cellular-resolution assessment of UCP2 expression independent of bulk tumour composition. Fifth, the TIDE-based prediction of immunotherapy resistance is derived from transcriptomic modelling rather than direct clinical immunotherapy response data; our cohort lacks prospective immunotherapy outcome records, and the predictive value of UCP2 for immunotherapy response requires validation in cohorts with documented treatment outcomes. Sixth, pseudotime trajectory analysis provides an inferred representation of TAM state transitions based on transcriptional similarity rather than direct longitudinal observation and should be interpreted as hypothesis-generating rather than definitive evidence of dynamic UCP2 regulation during TAM polarisation. Seventh, the proposed UCP2–lactate–TAM polarisation axis is supported by correlative transcriptomic evidence and existing literature but lacks direct metabolic validation within this cohort. Eighth, the pseudotime trajectory analysis was performed on TAM populations merged from three independent scRNA-seq datasets (GSE70630, GSE84465, and GSE89567) without formal batch effect correction; although dataset identity was retained and visualised to allow assessment of dataset mixing, residual batch effects cannot be entirely excluded, and findings should be interpreted with caution. Future studies incorporating metabolomic profiling or functional perturbation experiments would be required to establish this mechanistic link. Finally, while the present study focuses on glioma, the broader applicability of UCP2 as a TAM marker across other cancer types with distinct immune microenvironments warrants further investigation.

## Data Availability

The data that support the findings of this study are openly available in TCGA at https://portal.gdc.cancer.gov/, GTEx at https://gtexportal.org/, and GEO at https://www.ncbi.nlm.nih.gov/geo/. The institutional IHC data are available from the corresponding author upon reasonable request.

## Supplementary Materials

The supplementary material is available online at https://www.techscience.com/doi/10.32604/or.2026.082613/s1.
