# Supplementary material for: UCP2 Identifies Immunosuppressive Tumor-Associated Macrophages and Is Associated with Predicted Immunotherapy Resistance in Glioma
Source: Oncol Res. 2026 Jul 16;34(8):22. doi: 10.32604/or.2026.082613 (PMC13397320; doi:10.32604/or.2026.082613)
Supplement: Supplementary file 1 [file OncolRes-34-82613-s001.zip › TSP_OR_82613-s001_figures.docx]

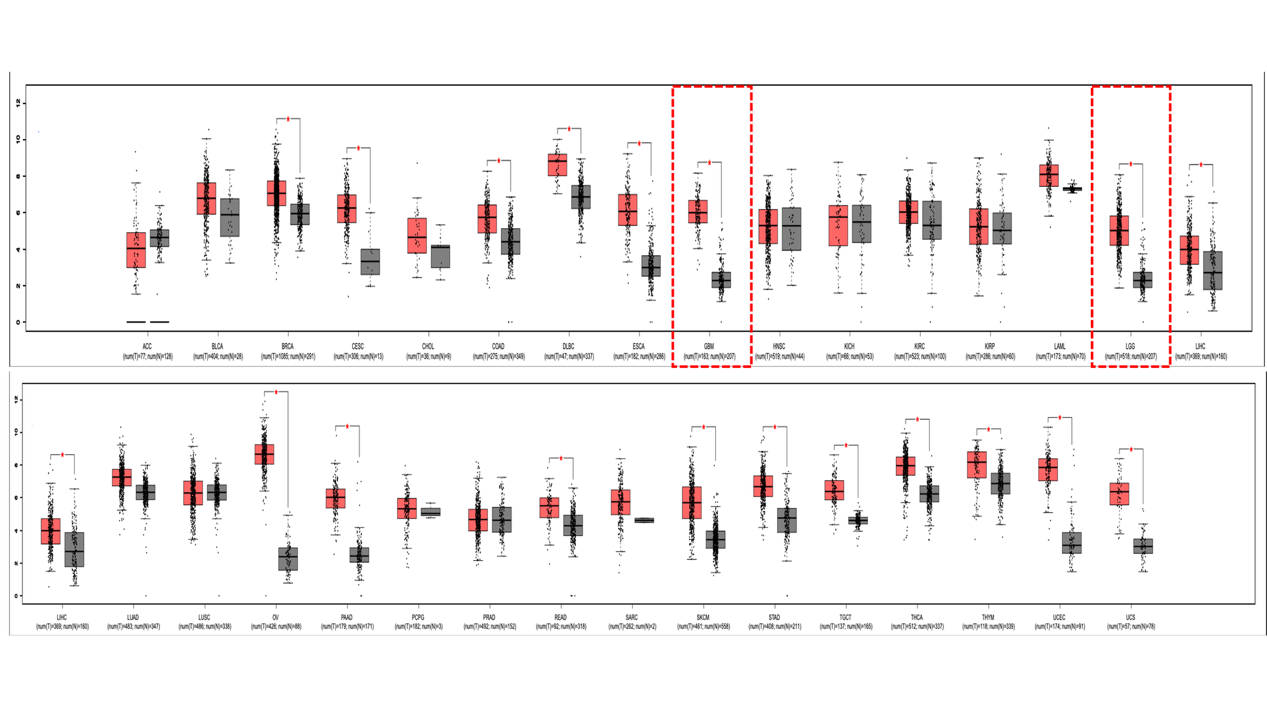


**
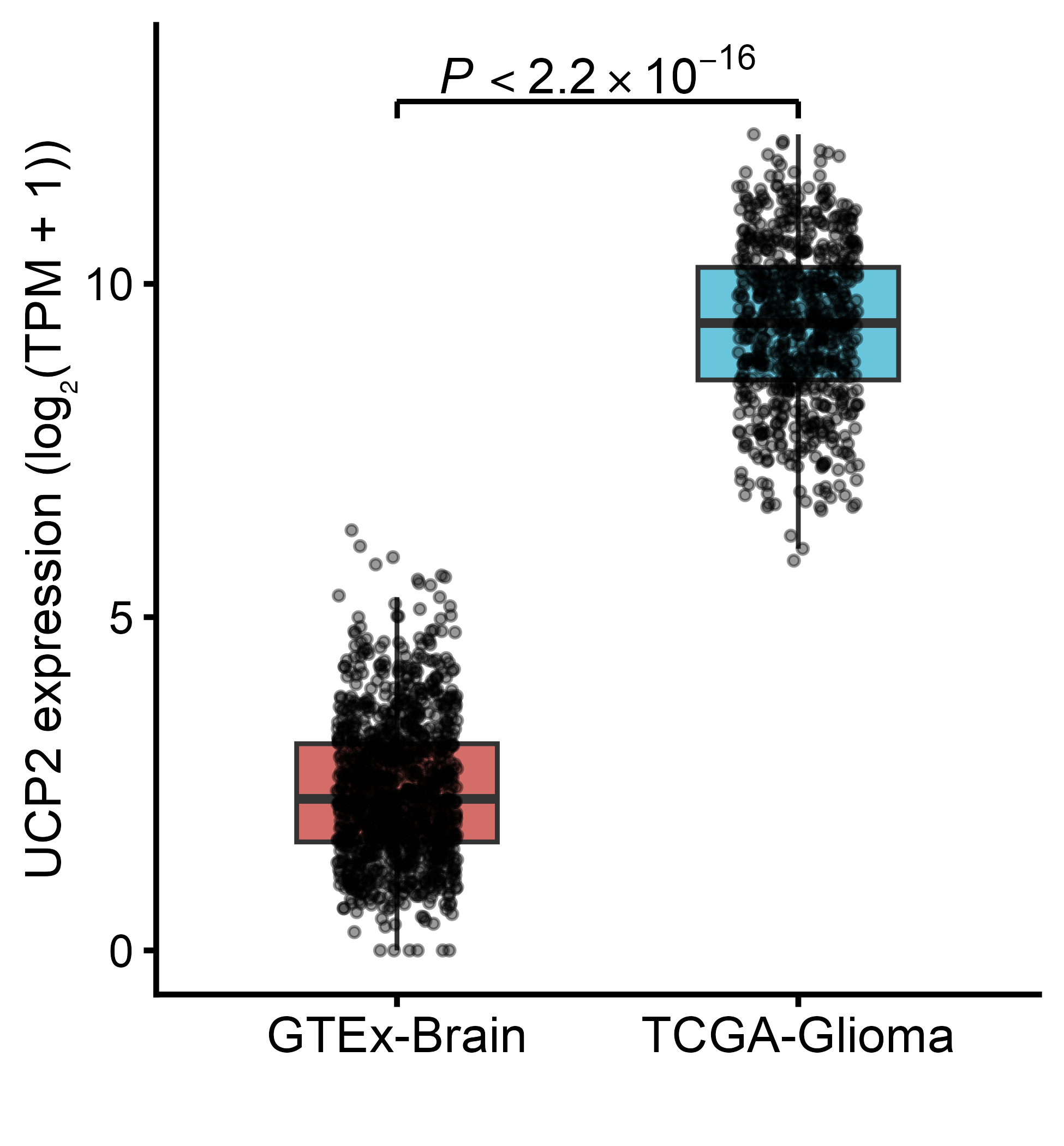
Supplementary Figure S1.** Pan-cancer expression profile of UCP2 across 31 TCGA cancer types based on GEPIA2.Box plots show UCP2 expression levels [log₂ (TPM + 1)] in tumor (red) and matched normal tissues (gray). Normal tissue data were derived from matched TCGA adjacent-normal samples and GTEx normal tissues as implemented in the GEPIA2 database. Asterisks indicate statistically significant differential expression (|log₂FC| > 1, p-value cutoff = 0.01). The red dashed box highlights the two glioma subtypes: low-grade glioma (LGG) and glioblastoma multiforme (GBM).

**Supplementary Figure S2**. UCP2 expression in TCGA glioma versus GTEx normal brain tissue.Box plots show UCP2 expression levels [log^₂^ (TPM + 1)] in GTEx normal brain tissue (n = 1,152; red) and TCGA glioma samples (n = 702; blue). Each dot represents an individual sample. Statistical significance was assessed using the Wilcoxon rank-sum test (P < 2.2×10⁻¹⁶).


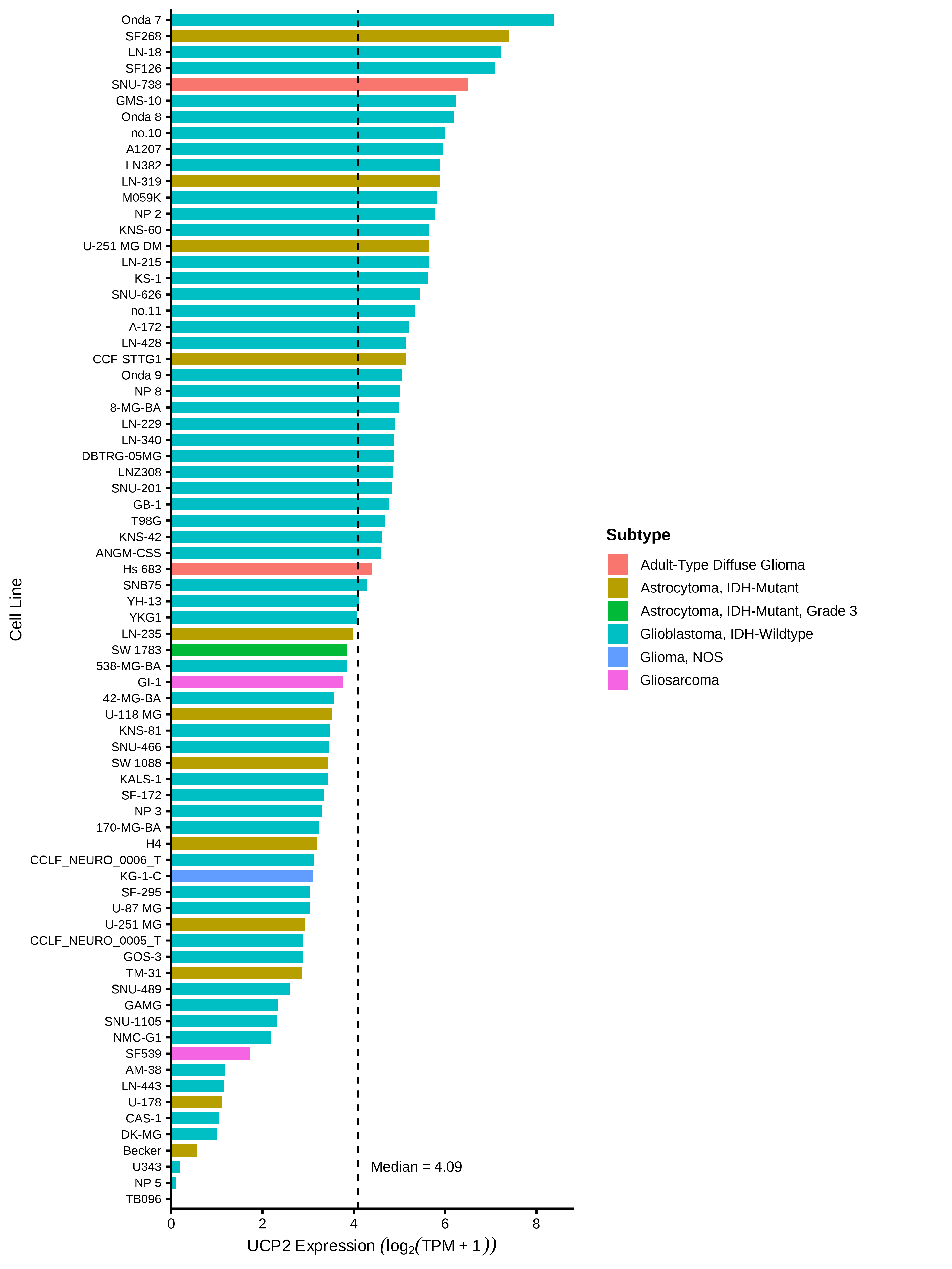
**Supplementary Figure S3**. UCP2 expression across glioma cell lines in the Cancer Cell Line Encyclopedia (CCLE). Bar plot showing UCP2 expression levels (log₂(TPM+1)) across 74 unique glioma cell lines derived from the CCLE dataset (DepMap portal, 24Q4 release). Cell lines are ordered by decreasing UCP2 expression along the y-axis. Bars are coloured by OncotreeSubtype classification as indicated in the legend. The dashed vertical line indicates the median UCP2 expression value (median = 4.09 log₂(TPM+1)). One cell line (TB096, Astrocytoma IDH-Mutant) showed no detectable UCP2 expression (value = 0).


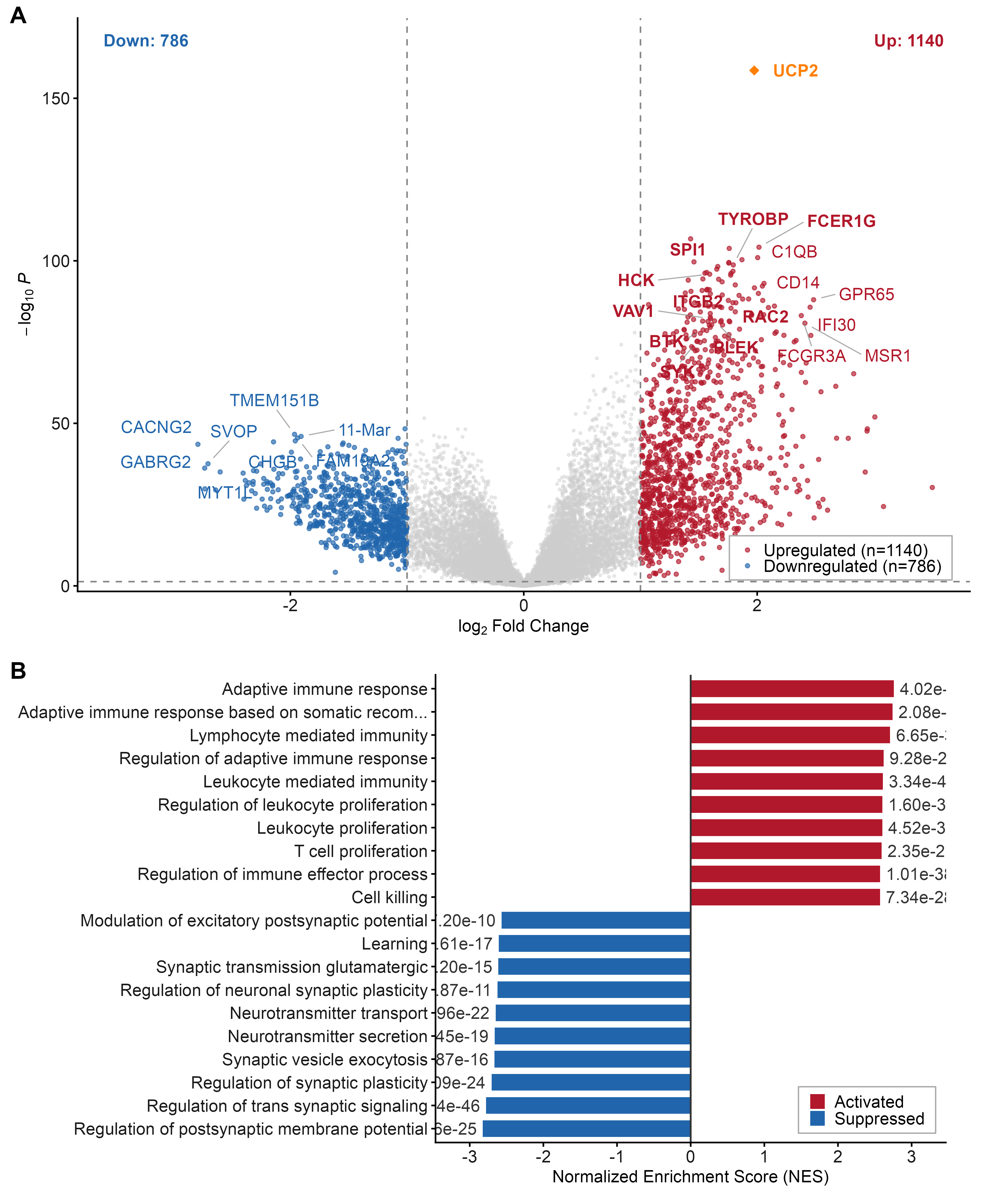
**Supplementary Figure S4**. Differential gene expression and gene set enrichment analysis between UCP2-high and UCP2-low glioma groups. (A) Volcano plot of differentially expressed genes (DEGs) between UCP2-high and UCP2-low TCGA glioma samples (n = 670), identified using the limma R package (|log2FC| > 1, FDR < 0.05). Red and blue dots indicate significantly upregulated and downregulated genes, respectively; grey dots represent non-significant genes. The orange diamond indicates UCP2. Selected hub genes (from PPI network analysis) and top statistically ranked genes are labelled. Dashed lines indicate thresholds of |log2FC| = 1 and FDR = 0.05 (−log10 P scale). (B) Bar plot of the top 10 activated and top 10 suppressed Gene Ontology Biological Process (GO BP) gene sets from Gene Set Enrichment Analysis (GSEA) of the full log2FC-ranked gene list (FDR < 0.05). Bars represent Normalised Enrichment Scores (NES); FDR-adjusted p values are annotated at bar ends.


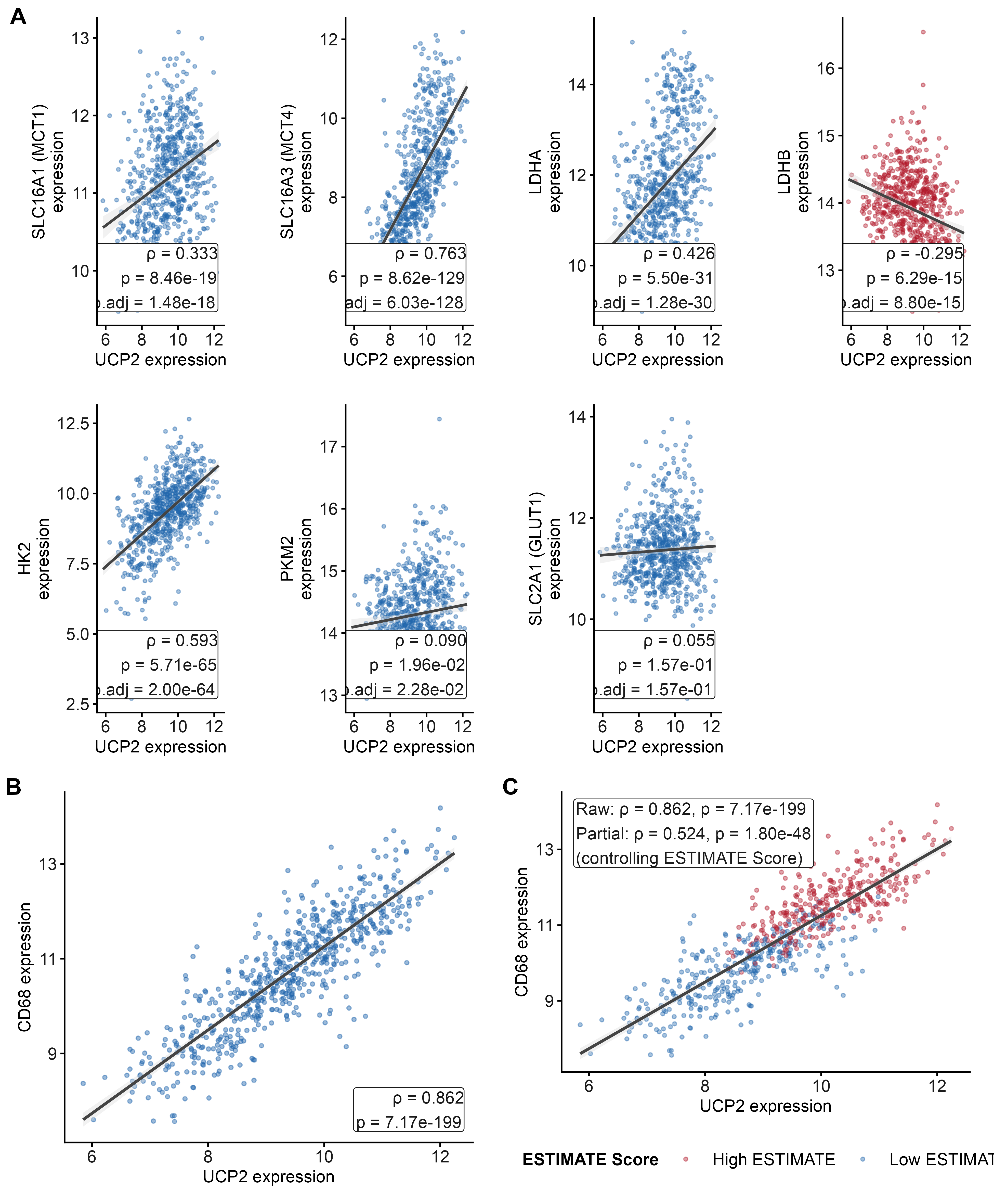
**Supplementary Figure S5**. UCP2 expression correlates with lactate metabolism-related genes and macrophage infiltration in glioma. **(A)** Scatter plots showing Spearman correlations between UCP2 and seven lactate metabolism-related genes across TCGA glioma samples (n = 670). Blue points indicate positive correlations; red points indicate negative correlations. Regression lines with 95% confidence intervals are shown. Spearman ρ, raw p-value, and Benjamini–Hochberg-adjusted p-value (p.adj) are displayed within each panel. **(B)** Scatter plot showing the Spearman correlation between UCP2 and CD68 mRNA expression across TCGA glioma samples (n = 670). The strong positive correlation (ρ = 0.862, p = 7.17×10⁻¹⁹⁹) confirms that the UCP2 transcriptomic signal in bulk glioma tissue is predominantly attributable to macrophage/microglial infiltration. **(C)** Scatter plot of UCP2 versus CD68 expression, with points coloured by ESTIMATE Score group (red, high ESTIMATE Score; blue, low ESTIMATE Score). Both raw Spearman correlation and partial Spearman correlation controlling for ESTIMATE Score are displayed. The persistence of a highly significant partial correlation (ρ = 0.524, p = 1.80×10⁻⁴⁸) confirms that the UCP2–CD68 association is independent of tumour purity variation.


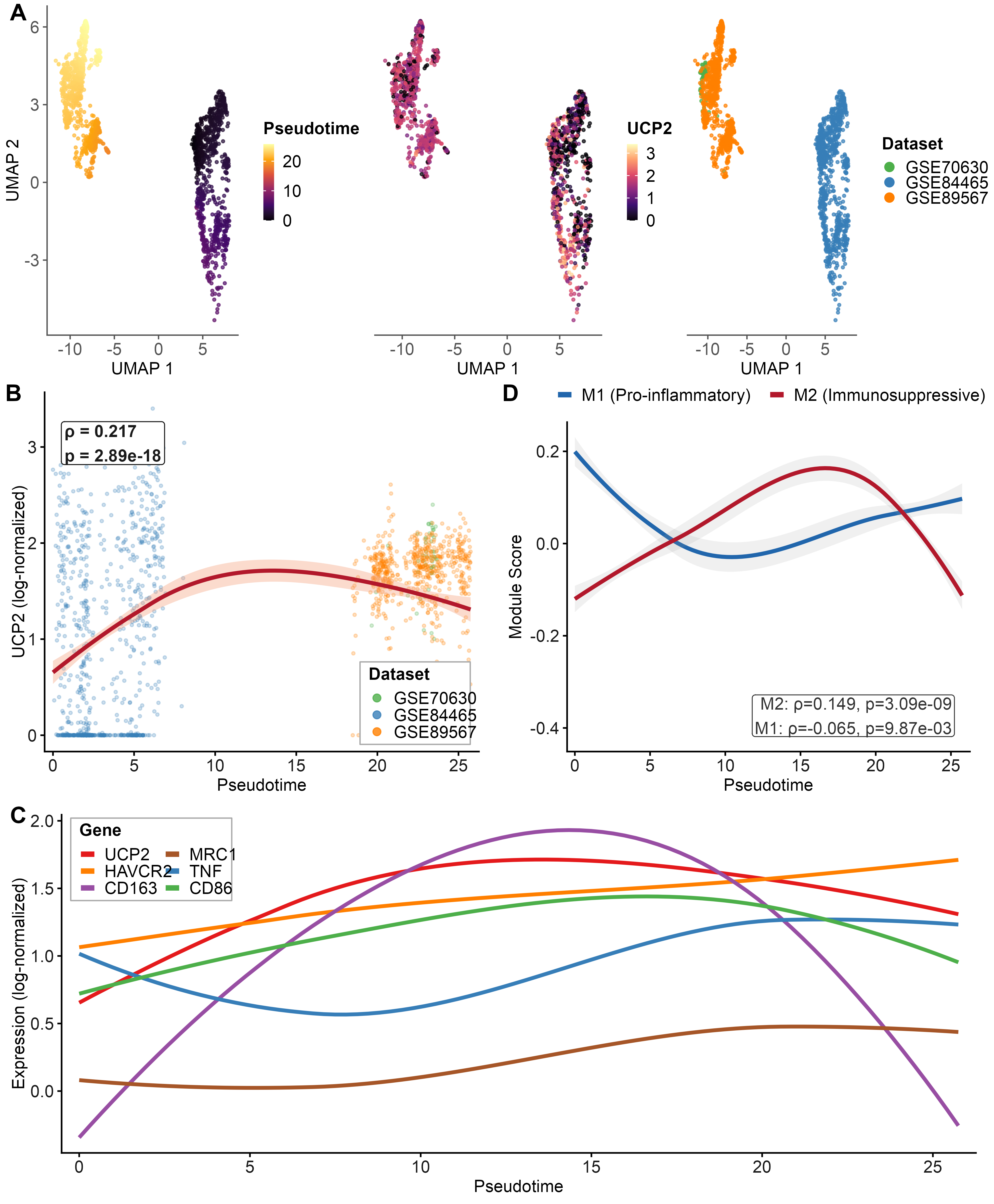
**Supplementary Figure S6**. UCP2 expression progressively increases along the tumour-associated macrophage (TAM) immunosuppressive pseudotime trajectory.(A) UMAP embedding of the pooled TAM population (n = 1,575) coloured by inferred Slingshot pseudotime value.(B) UCP2 expression plotted against pseudotime across three scRNA-seq cohorts (coloured by dataset); loess regression line with 95% CI shown (Spearman rho = 0.217, p = 2.89e-18).(C) Expression dynamics of immunosuppressive markers (UCP2,HAVCR2, CD163, MRC1) and pro-inflammatory markers (TNF,CD86) along pseudotime.(D) M1 and M2 module scores along pseudotime. M2 increases (rho = 0.149, p = 3.09e-09); M1 decreases (rho = -0.065, p = 0.0099).
